# Supplementary material for: Malaria parasites require a divergent heme oxygenase for apicoplast gene expression and biogenesis
Source: eLife. 2024 Dec 11;13:RP100256. doi: 10.7554/eLife.100256 (PMC11634067; doi:10.7554/eLife.100256)
Supplement: Figure 1—figure supplement 3—source data 1. [file elife-100256-fig1-figsupp3-data1.docx]

**Data collection and refinement statistics.**

Statistical values given in parenthesis refer to the highest resolution bin.

| Data Collection | **WT** | **SeMet** |
| --- | --- | --- |
| Space Group | P 2_1_ 2_1_ 2_1_ | P 2_1_ 2_1_ 2_1_ |
| Unit cell |  |  |
| a, b, c (Å) | 54.91, 55.37, 162.40 | 55.37, 55.64, 164.64 |
| Angles (°) | α = β = γ = 90 | α = β = γ = 90 |
| Resolution (Å) | 55.4 – 2.78 (2.93 – 2.78) | 31.9 – 2.65 (2.79 – 2.65) |
| Wavelength | 1.0000 | 0.97921 |
| Observations | 85,682 (11,778) | 128,208 (18,962) |
| Unique reflections | 13,064 (1,843) | 15,379 (2,206) |
| *R_merge_* | 10.7 (0.715) | 0.084 (0.614) |
| *R_p.i.m._***^a^** | 0.045 (0.303) | 0.044 (0.323) |
| *I / σ (I)* | 11.2 (2.3) | 14.2 (3.1) |
| Multiplicity | 6.6 (6.4) | 4.5 (8.6)**^a^** |
| Completeness (%) | 99.8 (99.5) | 99.7 (100)**^a^** |
| Refinement Statistics |  |  |
| Resolution (Å) | 55.4 − 2.78 |  |
| *R_work_* / *R_free_* (%)^b^ | 20.3 / 26.4 |  |
| No. protein chains | 2 |  |
| No. atoms |  |  |
| Protein | 3,083 |  |
| Other (not solvent) | 10 |  |
| Water | 15 |  |
| B-factor (Å^2^) |  |  |
| Protein | 70.0 |  |
| Other (not solvent) | 52.2 |  |
| Water | 50.7 |  |
| Ramachandran Plot |  |  |
| Preferred (%) | 93.4 |  |
| Allowed (%) | 6.6 |  |
| Outliers (%) | 0 |  |
| RMSD Bond (Å) | 0.009 |  |
| RMSD Angle (°) | 1.56 |  |
| PDB identification code | 7ABC |  |

**^a^** Calculated for equivalent reflections (within I^+^ or I^-^).
