# Supplementary figures and images for: Malaria parasites require a divergent heme oxygenase for apicoplast gene expression and biogenesis
Source: eLife. 2024 Dec 11;13:RP100256. doi: 10.7554/eLife.100256 (PMC11634067; doi:10.7554/eLife.100256)

Labeled blot

Unlabeled raw blot

PfHO-GFP

Untreated Dox/IPP

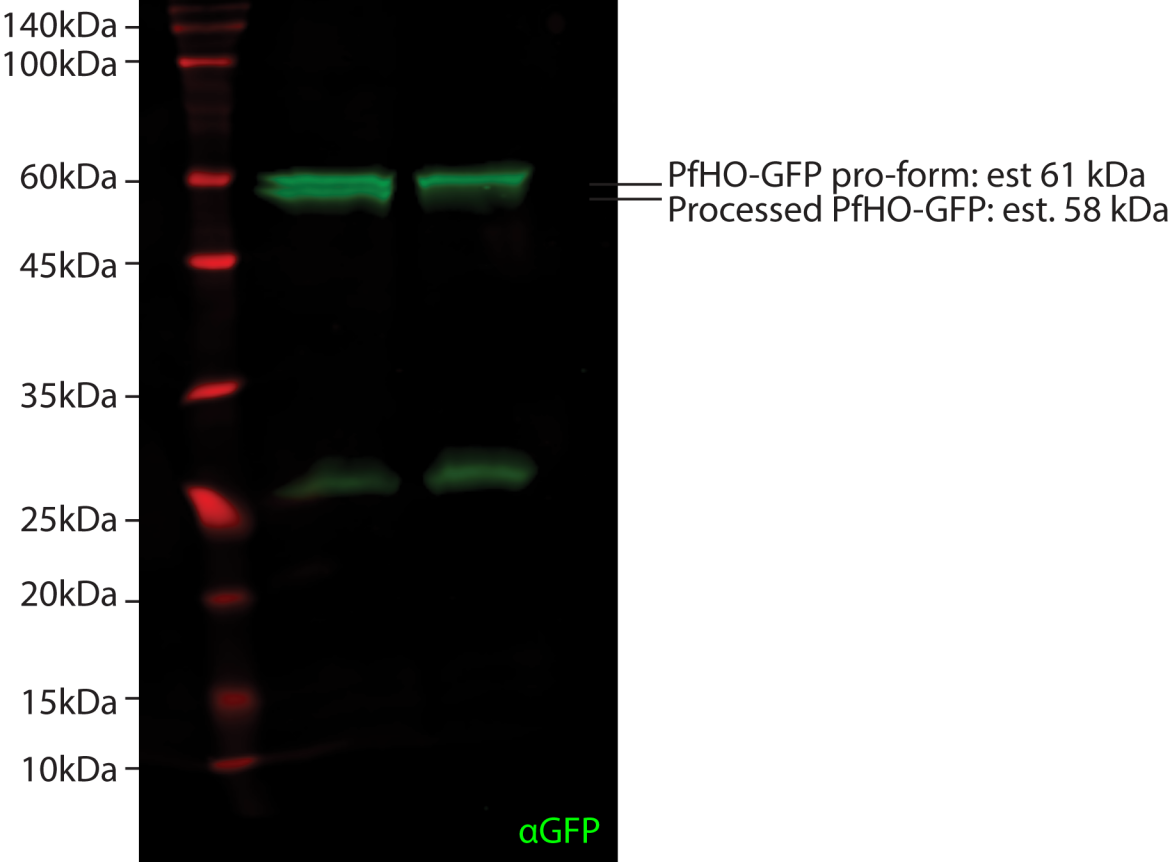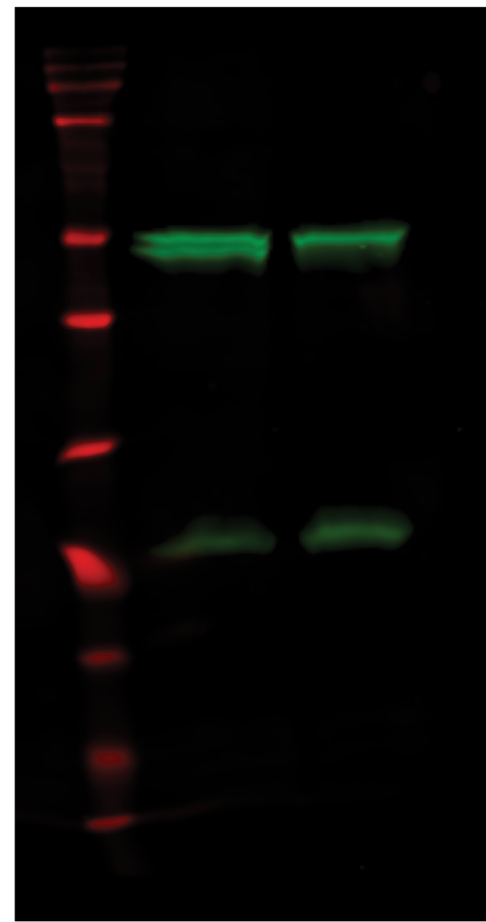

Supplement: Figure 2—source data 1. [file elife-100256-fig2-data1.zip › figure 2 - source data 1 - PfHOGFP WB.pdf]

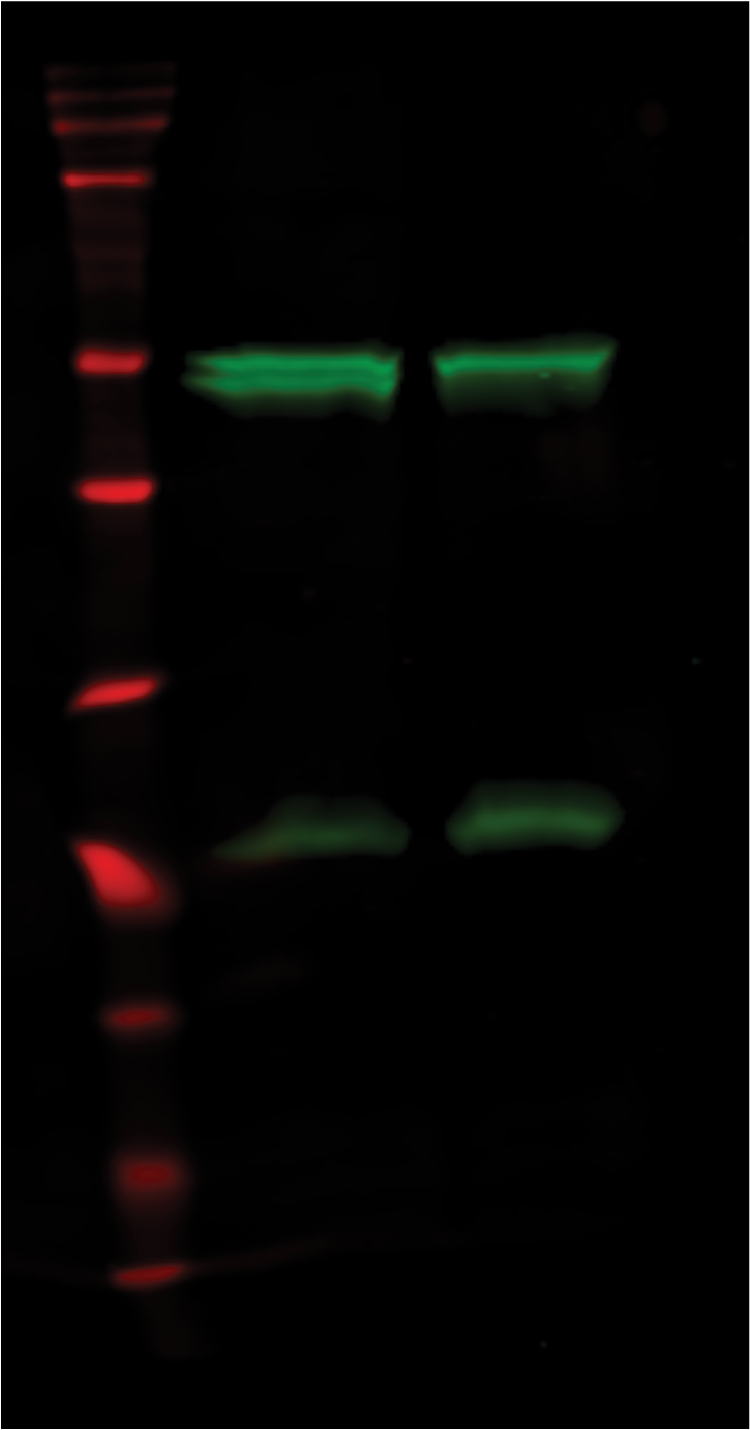

Supplement: Figure 2—source data 2. [file elife-100256-fig2-data2.zip › figure 2 - source data 2 - Figure 2B.tif]

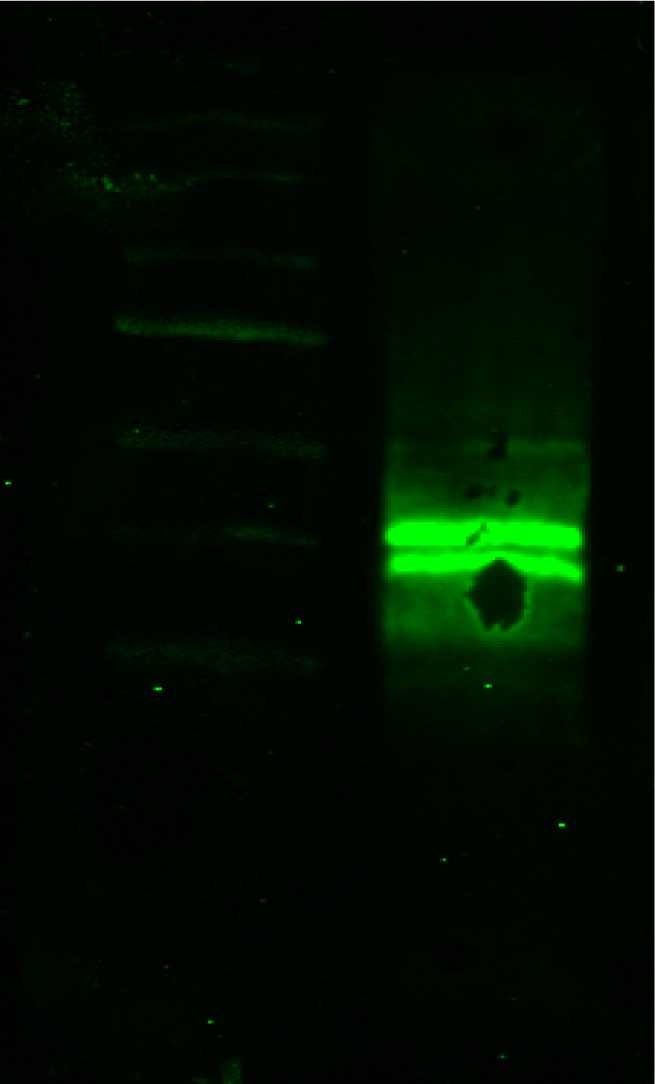

Supplement: Figure 2—source data 4. [file elife-100256-fig2-data4.zip › Figure 2 - source data 4 for Figure 2E/figure 2 - source data 4 - Figure 2E G.tif]

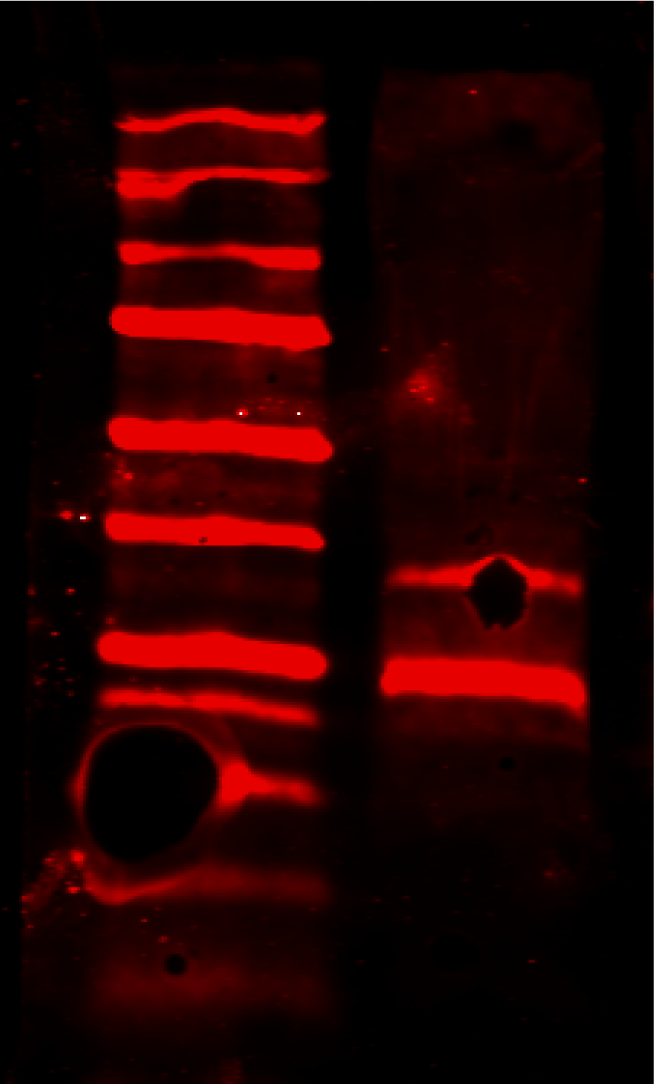

Supplement: Figure 2—source data 4. [file elife-100256-fig2-data4.zip › Figure 2 - source data 4 for Figure 2E/figure 2 - source data 4 - Figure 2E R.tif]

# Labeled blot

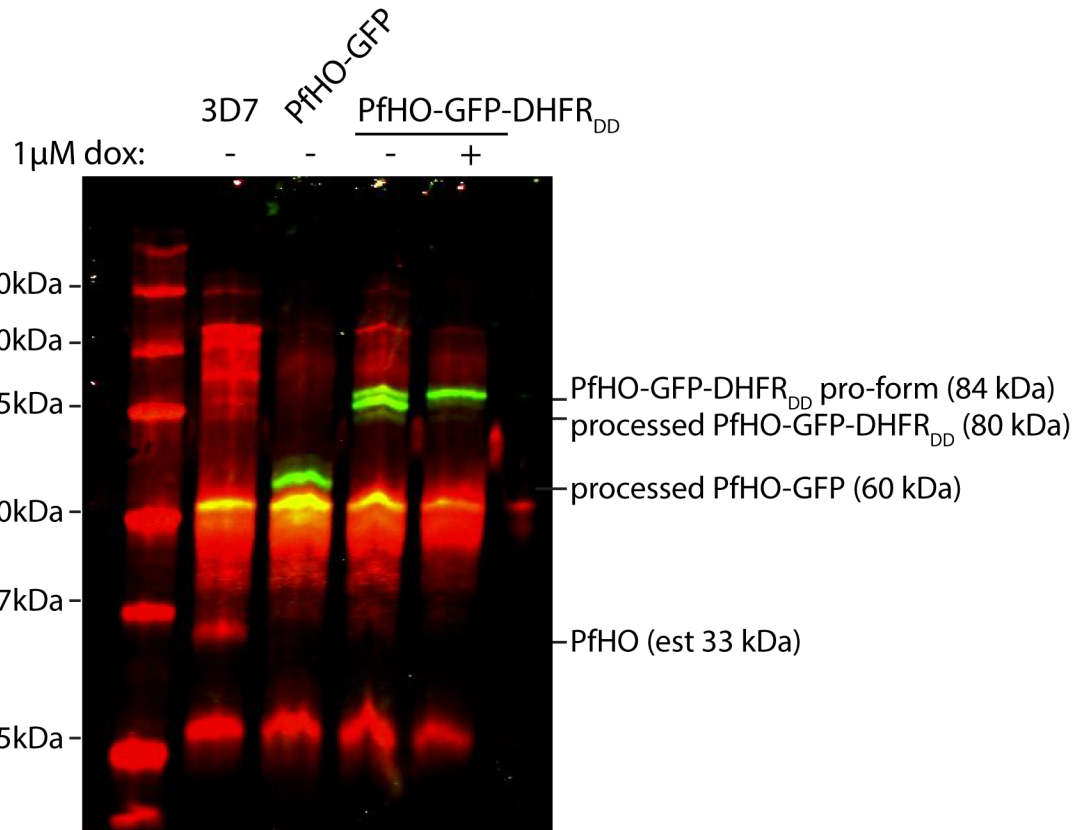

# Unlabeled raw blots

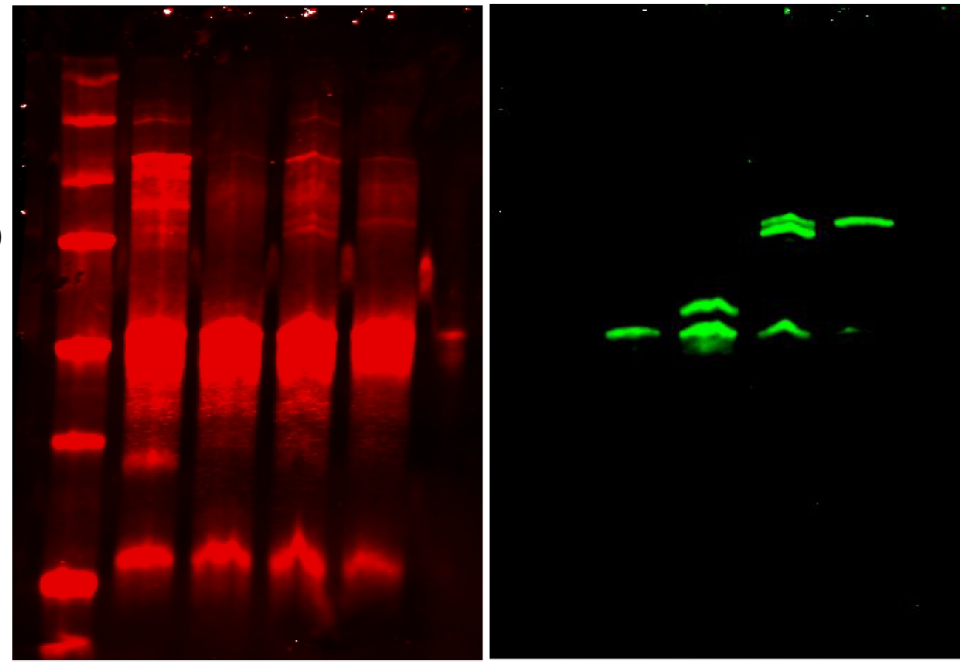

Supplement: Figure 3—source data 1. [file elife-100256-fig3-data1.zip › figure 3 - source data 1 - PfHO-GDB WB.pdf]

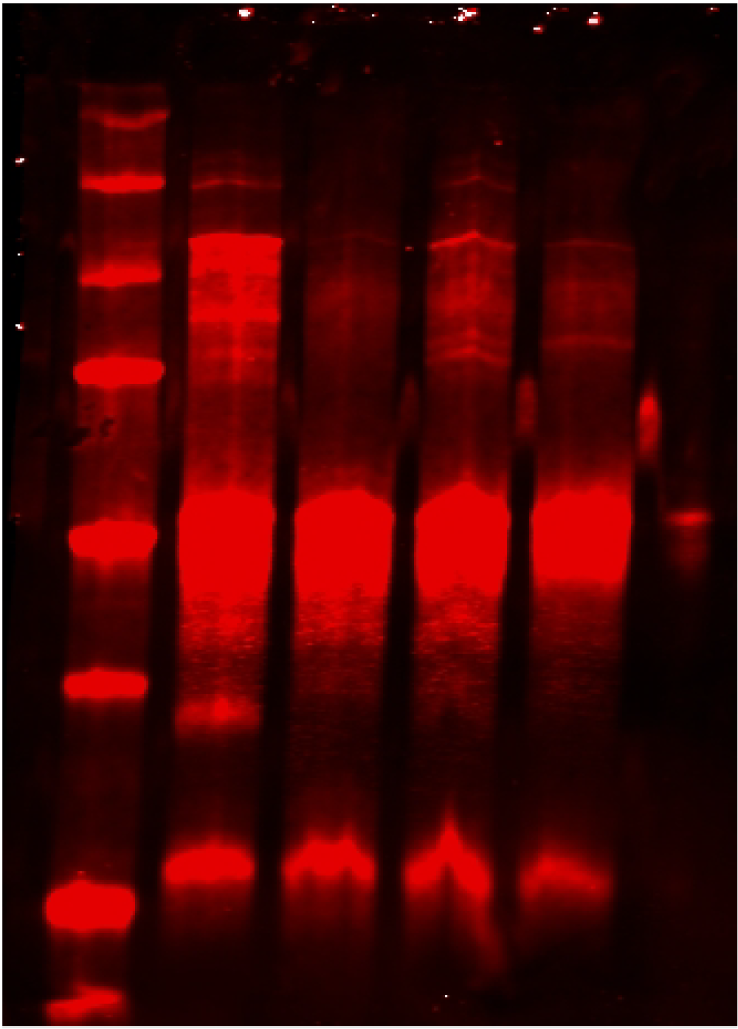

Supplement: Figure 3—source data 2. [file elife-100256-fig3-data2.zip › figure 3 - source data 2/figure 3 - source data 1 - PfHO-GDB WB unlabeled raw R.tif]

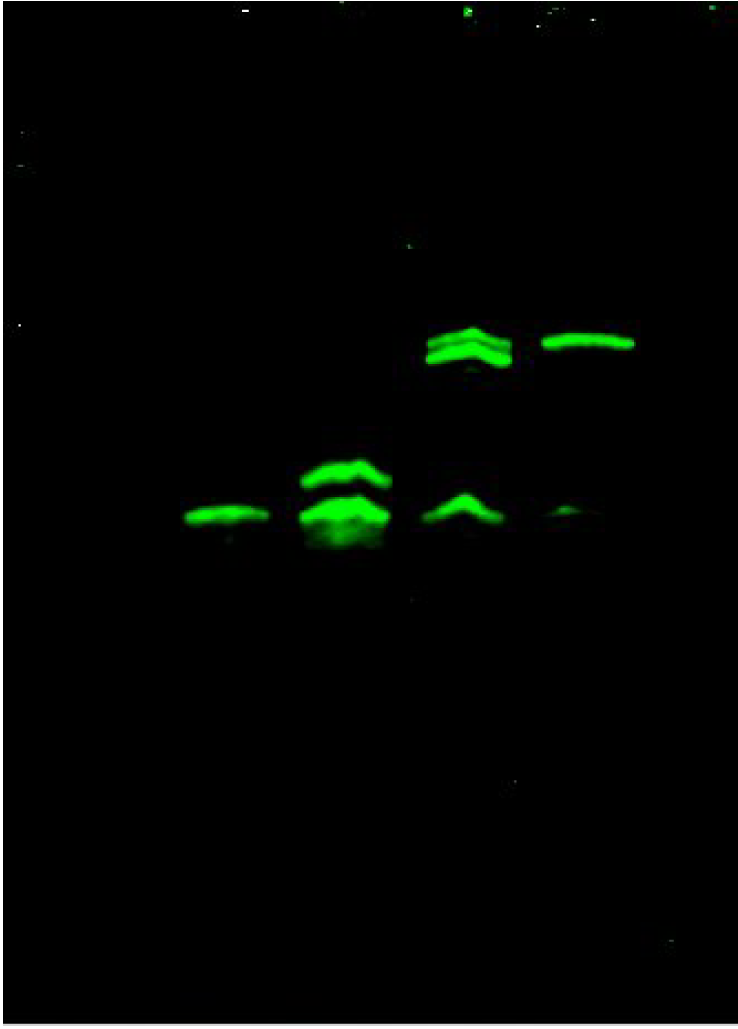

Supplement: Figure 3—source data 2. [file elife-100256-fig3-data2.zip › figure 3 - source data 2/figure 3 - source data 1 - PfHO-GDB WB unlabeled raw G.tif]

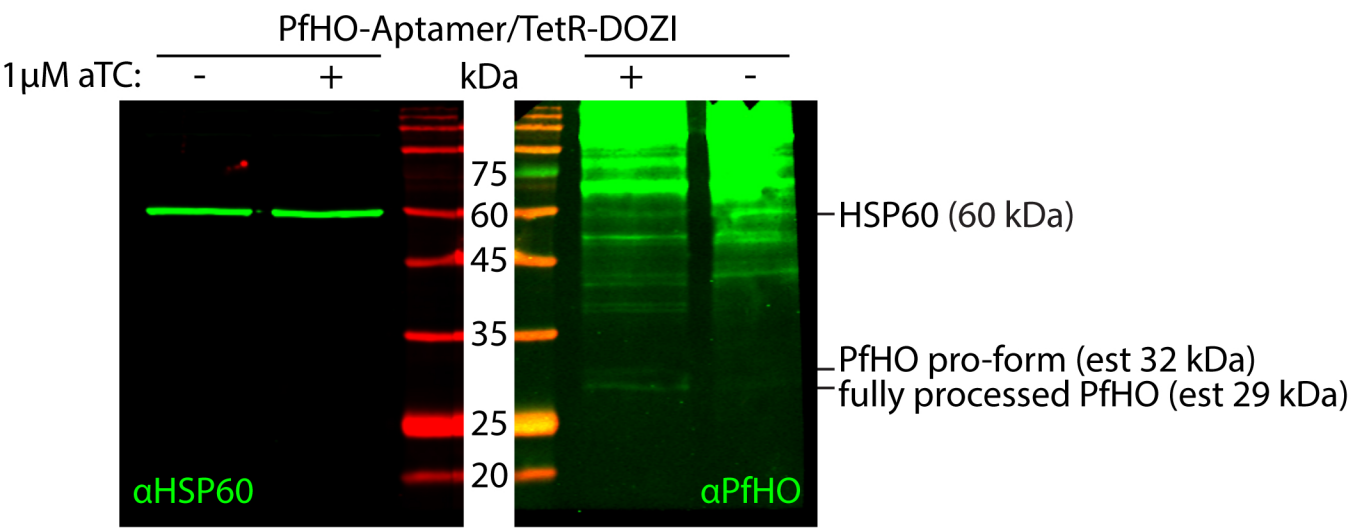

Supplement: Figure 3—source data 3. [file elife-100256-fig3-data3.zip › figure 3 - source data 3.pdf]

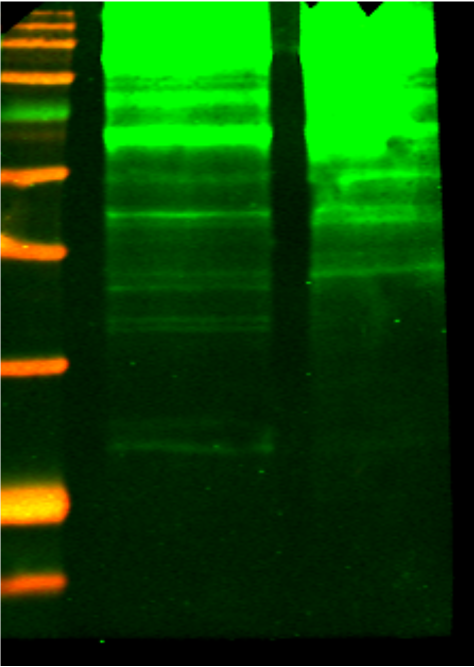

Supplement: Figure 3—source data 4. [file elife-100256-fig3-data4.zip › figure 3 - source data 4/figure 3 - source data 2 - PfHO apt WB unlabeled raw 2.tif]

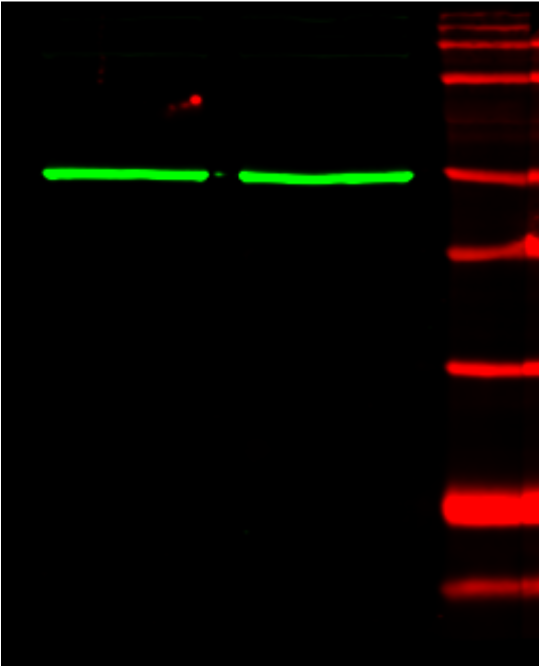

Supplement: Figure 3—source data 4. [file elife-100256-fig3-data4.zip › figure 3 - source data 4/figure 3 - source data 2 - PfHO apt WB unlabeled raw 1.tif]

Labeled blot

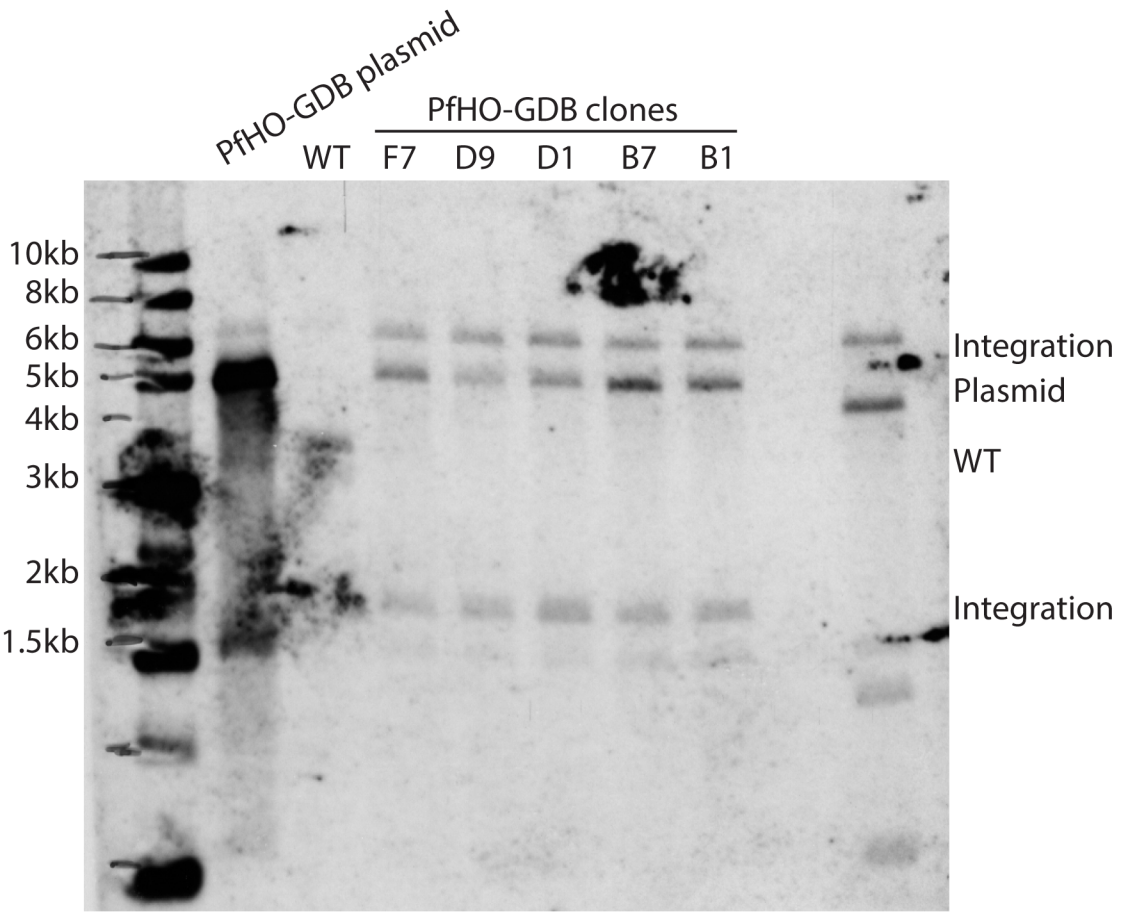

Unlabeled raw blot

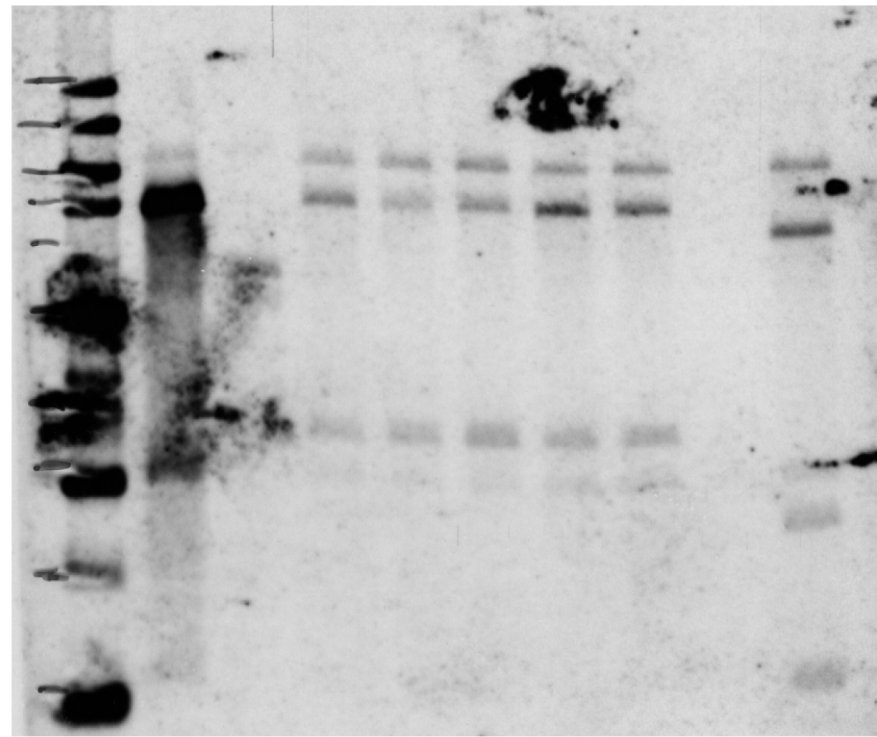

Supplement: Figure 3—figure supplement 1—source data 1. [file elife-100256-fig3-figsupp1-data1.zip › figure 3 - source data 5 - PfHO-GDB SB.pdf]

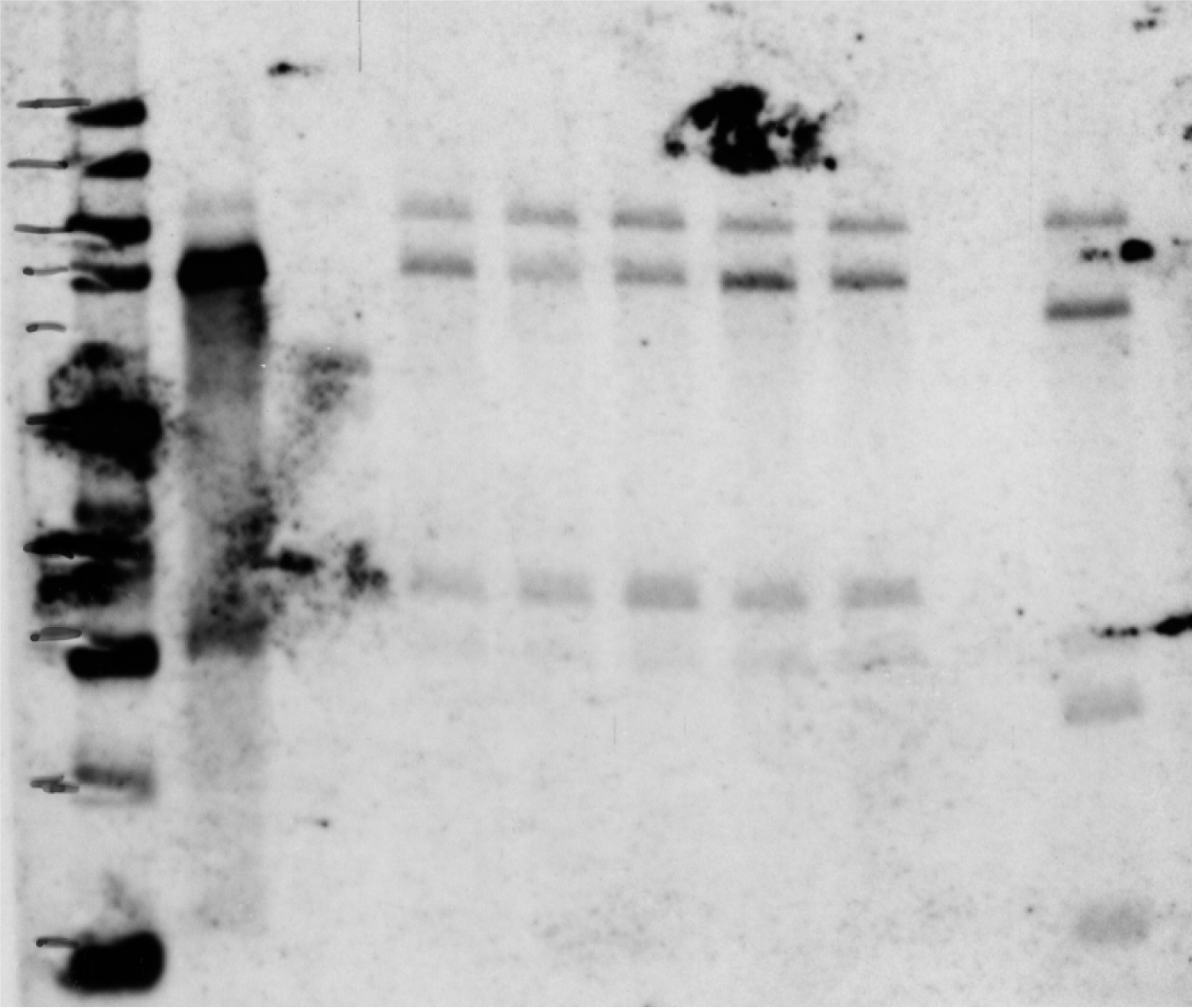

Supplement: Figure 3—figure supplement 1—source data 2. [file elife-100256-fig3-figsupp1-data2.zip › figure 3 - source data 6 - PfHO-GDB SB unlabeled raw.tif]

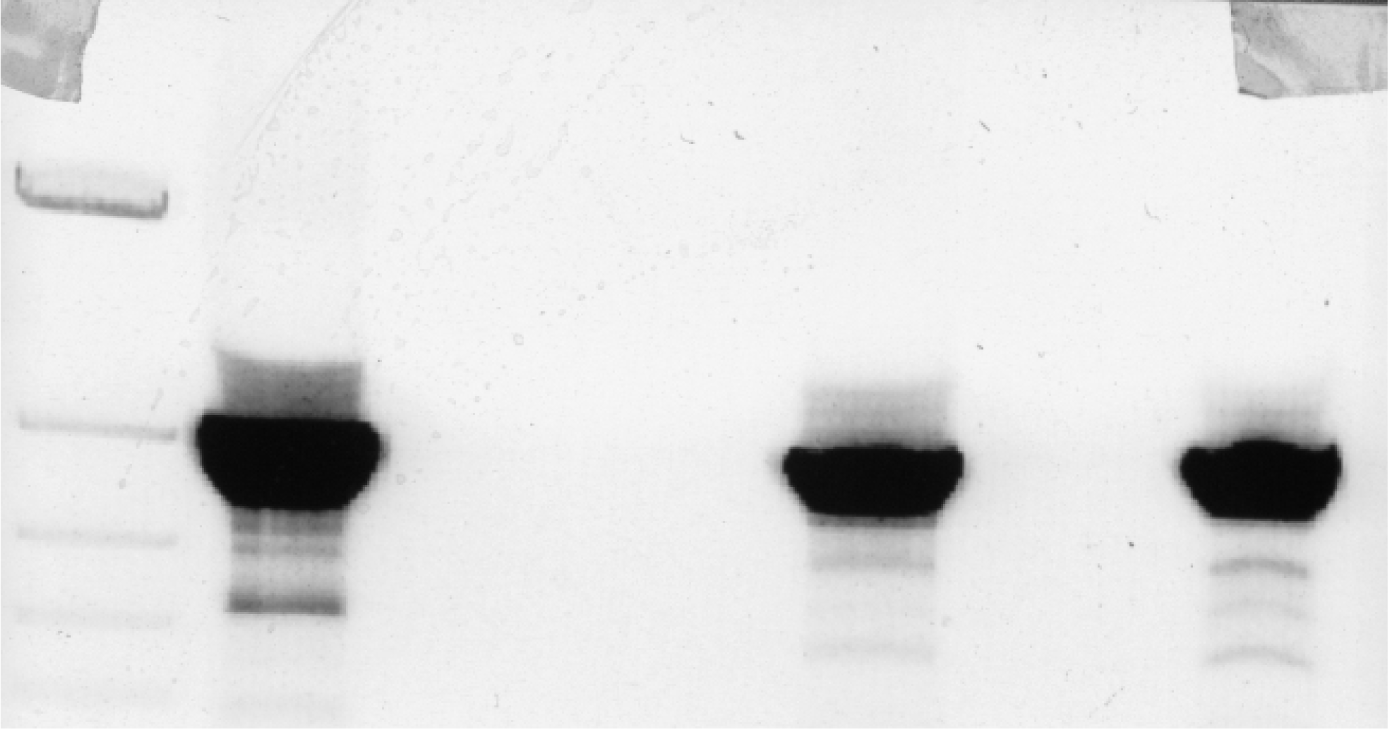

Supplement: Figure 3—figure supplement 1—source data 4. [file elife-100256-fig3-figsupp1-data4.zip › figure 3 - source data 8 - PfHO-glmS PCR gel unlabeled raw.tif]

## Labeled gel

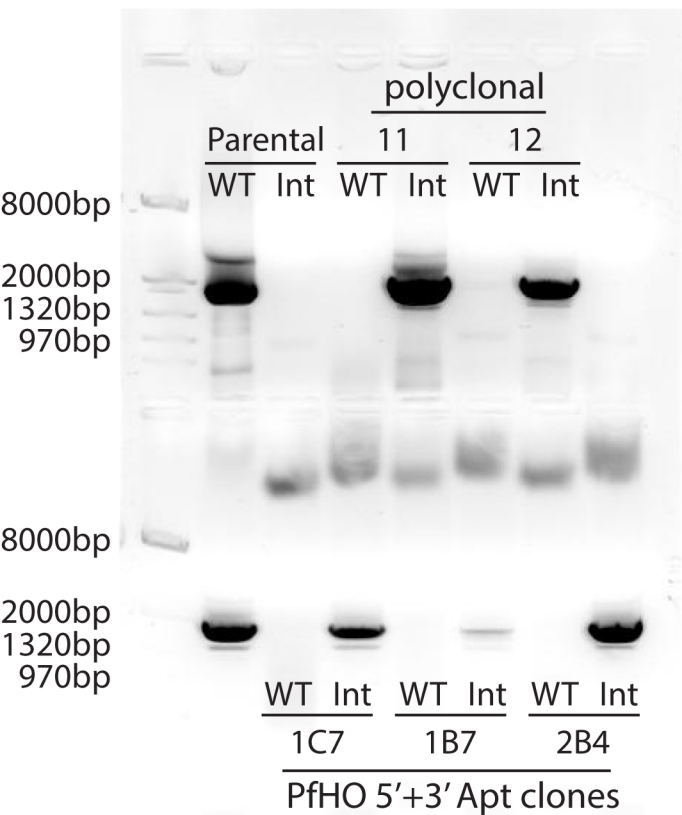

## Unlabeled raw gel

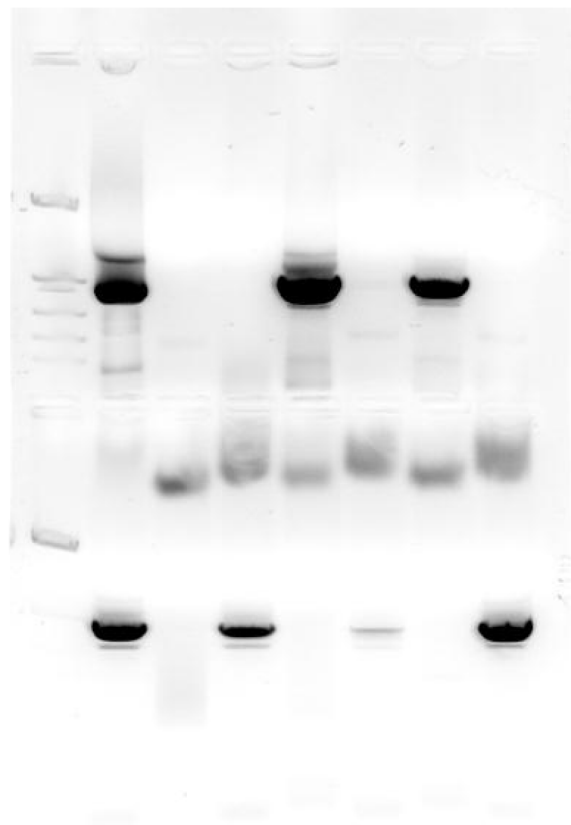

Supplement: Figure 3—figure supplement 4—source data 1. [file elife-100256-fig3-figsupp4-data1.zip › figure 3 - source data 9 - PfHO-Apt PCR gel.pdf]

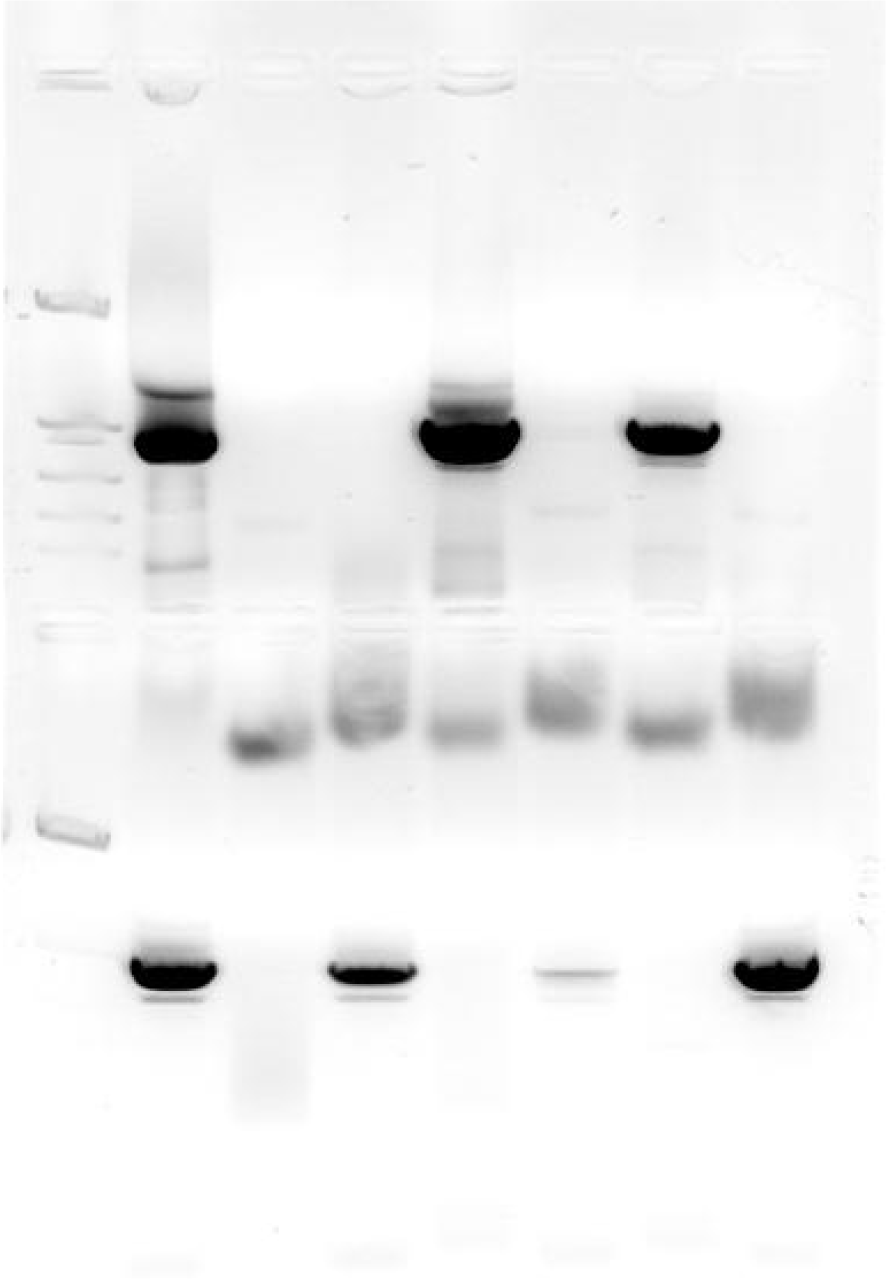

Supplement: Figure 3—figure supplement 4—source data 2. [file elife-100256-fig3-figsupp4-data2.zip › figure 3 - source data 10 - PfHO-Apt PCR gel unlabeled raw.tif]

Labeled blot

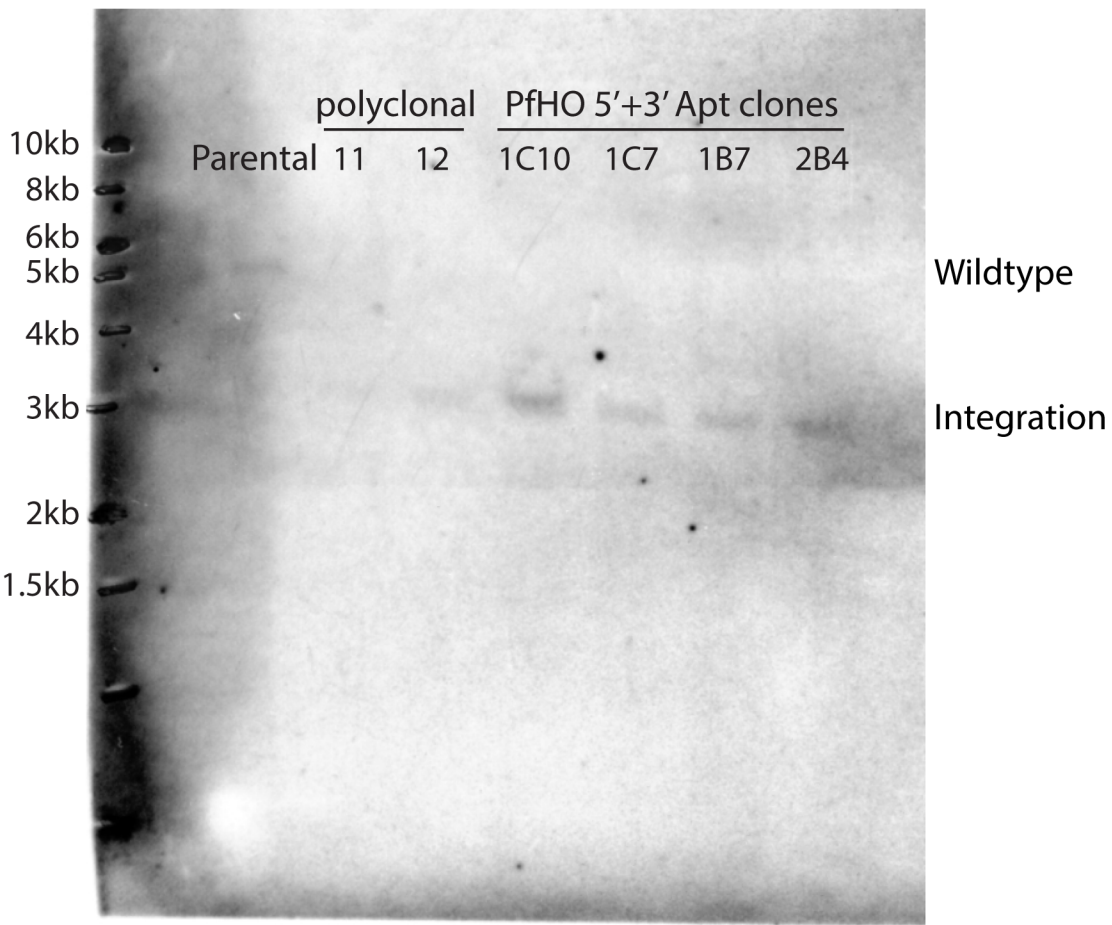

Unlabeled raw blot

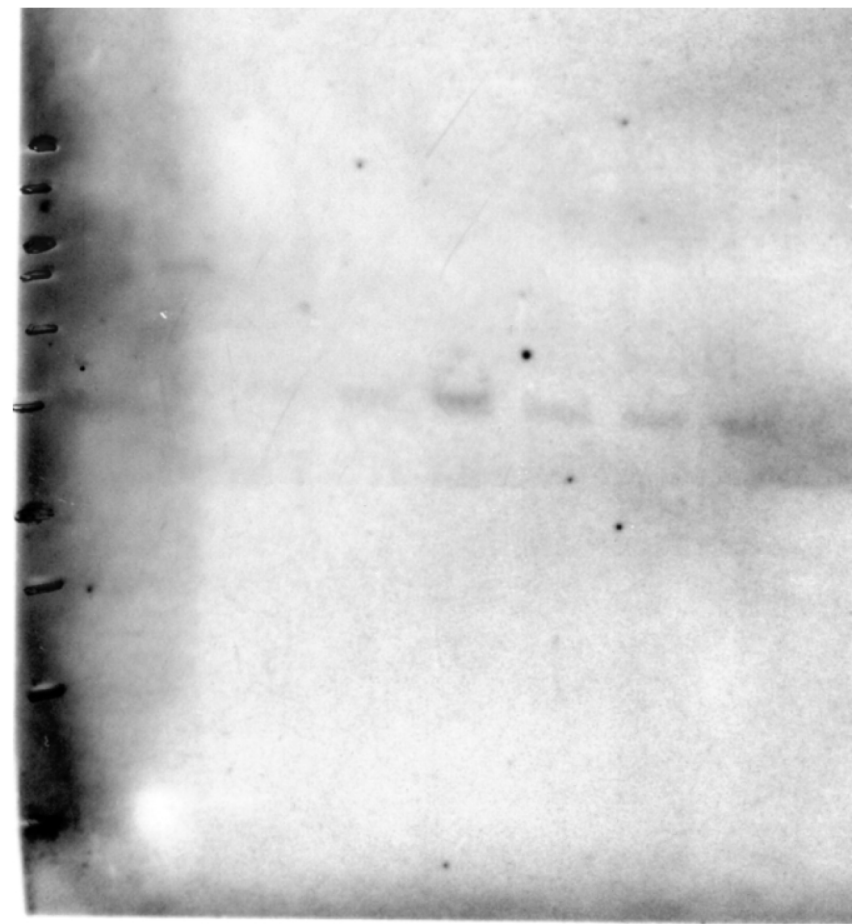

Supplement: Figure 3—figure supplement 4—source data 3. [file elife-100256-fig3-figsupp4-data3.zip › figure 3 - source data 11 - PfHO-Apt SB.pdf]

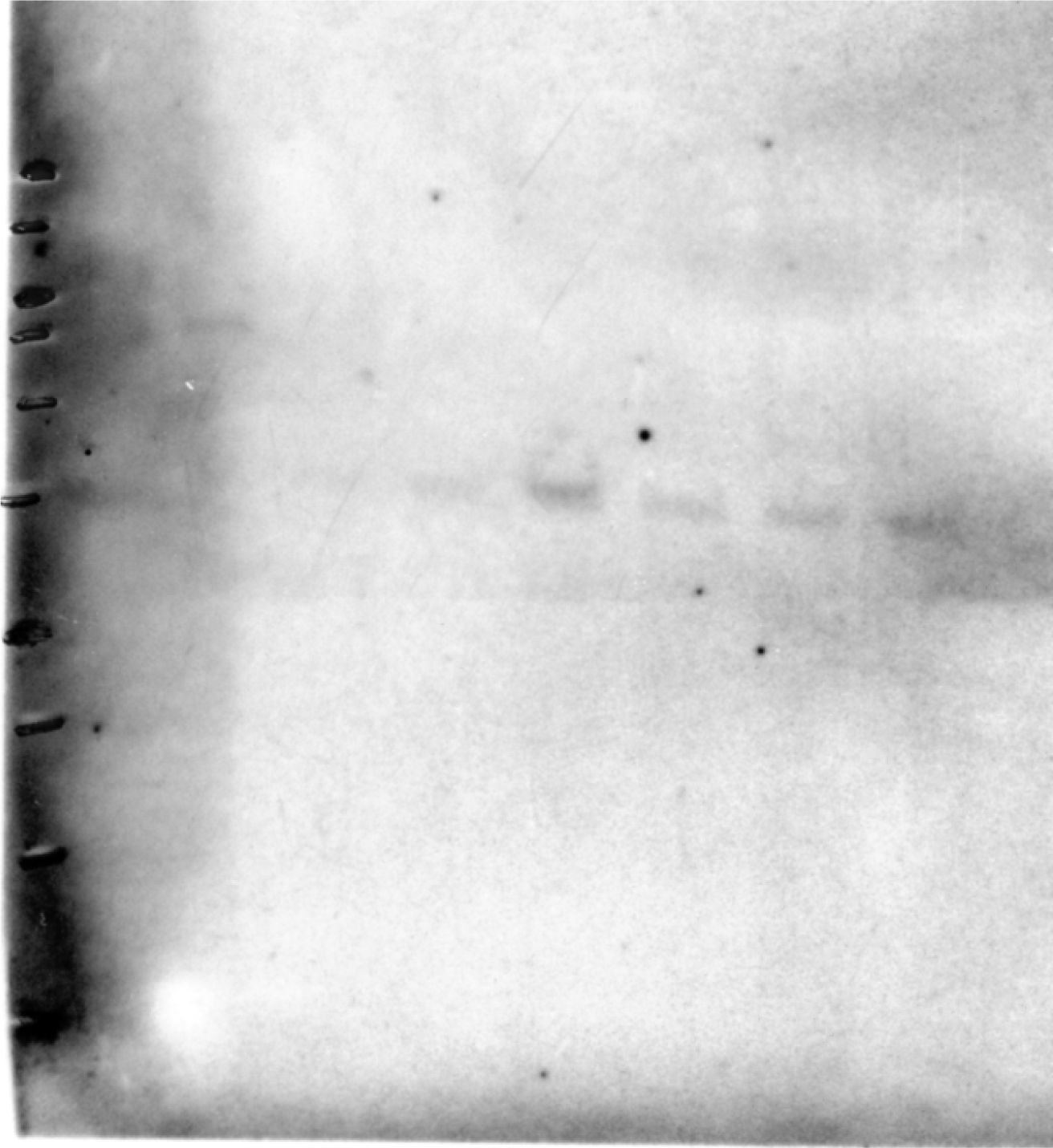

Supplement: Figure 3—figure supplement 4—source data 4. [file elife-100256-fig3-figsupp4-data4.zip › figure 3 - source data 12 - PfHO-Apt SB unlabeled raw.tif]

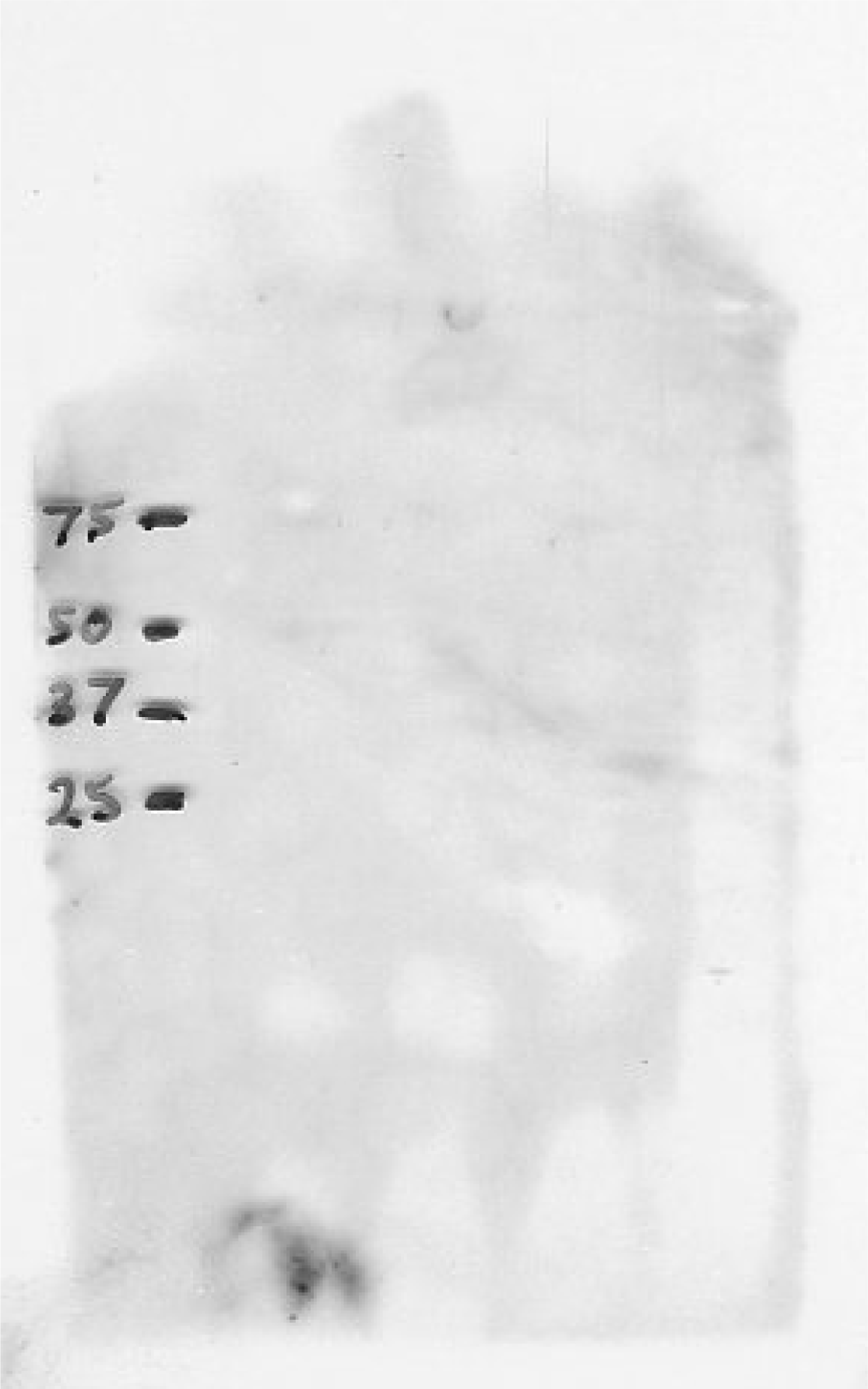

Supplement: Figure 3—figure supplement 5—source data 1. [file elife-100256-fig3-figsupp5-data1.zip › figure 3 - source data 14 - PfHO ab prebleed unlabeled raw.tif]

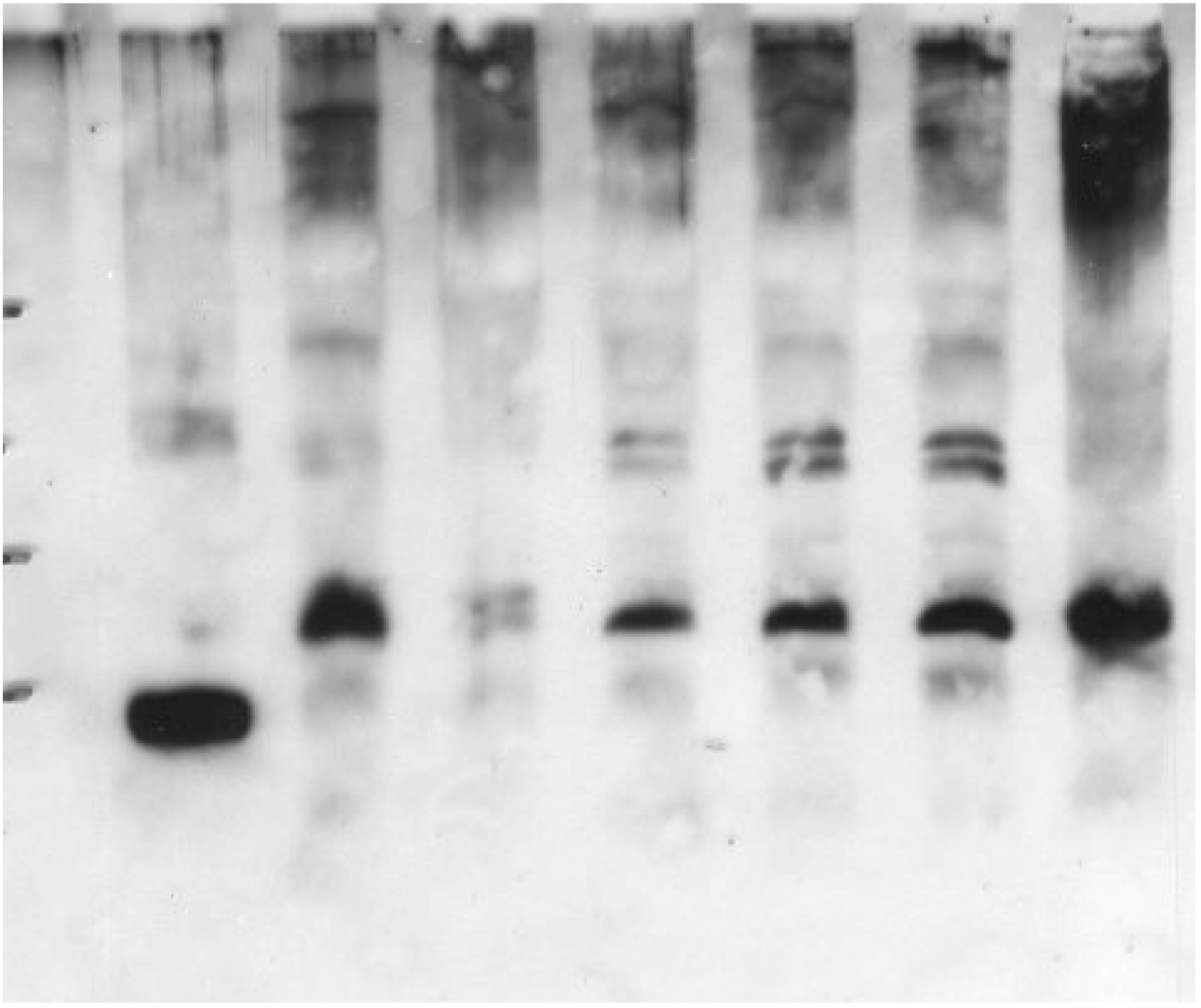

Supplement: Figure 3—figure supplement 5—source data 3. [file elife-100256-fig3-figsupp5-data3.zip › figure 3 - source data 16 - PfHO ab final bleed unlabeled raw.tif]

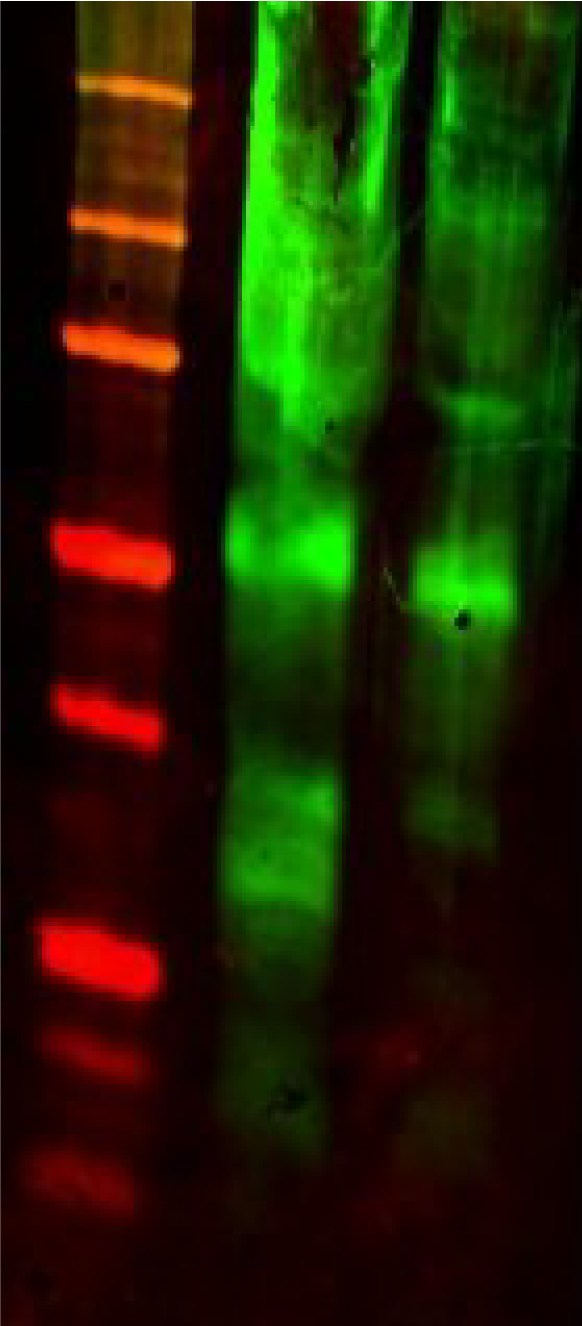

Supplement: Figure 3—figure supplement 6—source data 2. [file elife-100256-fig3-figsupp6-data2.zip › figure 3 - source data 18 - PfHO apt WB unlabeled raw.tif]

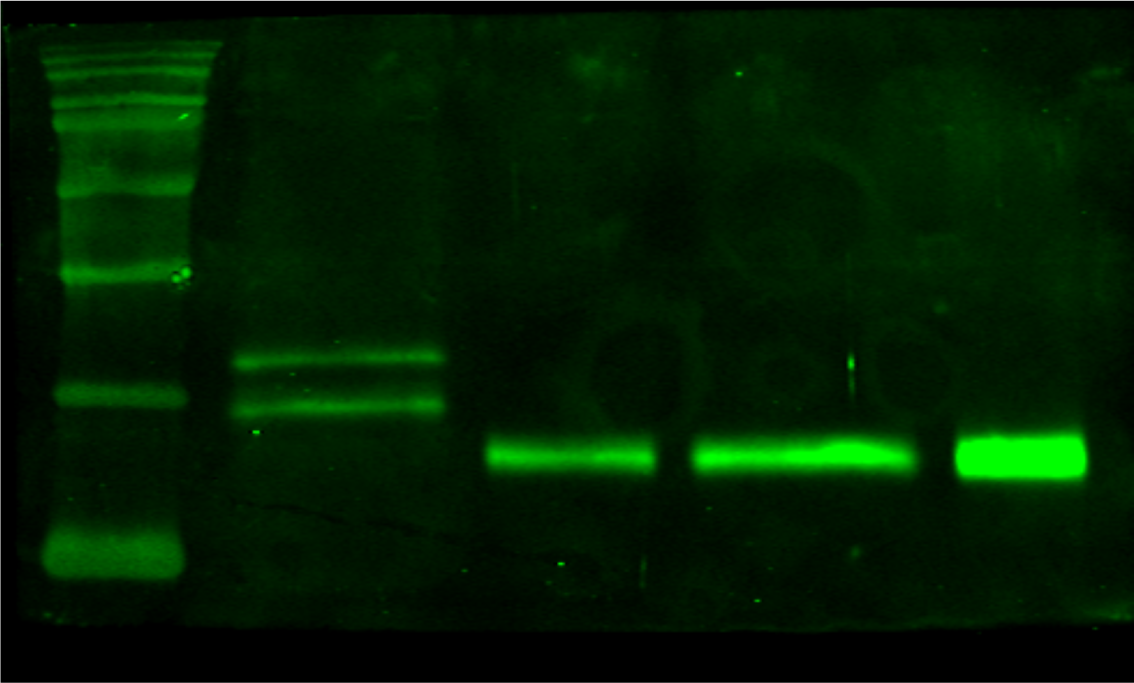

Supplement: Figure 4—source data 2. [file elife-100256-fig4-data2.zip › figure 4 - source data 1 - processing WB unlabeled raw G.tif]

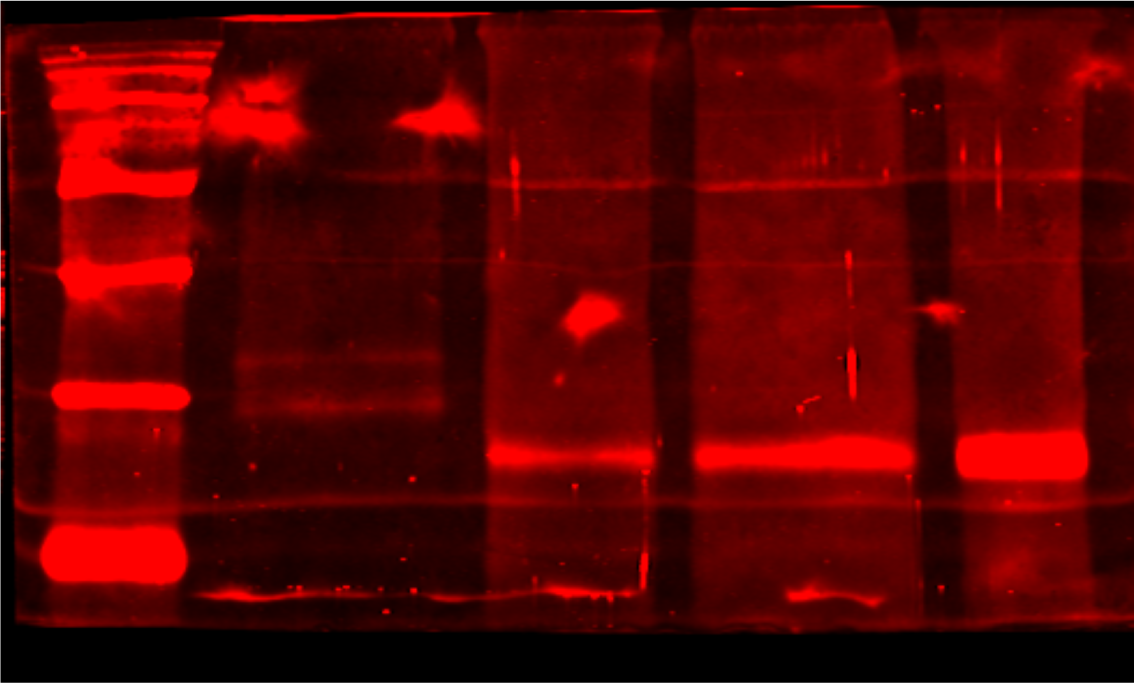

Supplement: Figure 4—source data 2. [file elife-100256-fig4-data2.zip › figure 4 - source data 1 - processing WB unlabeled raw R.tif]

Labeled blot

1 $\mu$ M aTC:      PfHO-GFP  
                          +      -      ACPL-HO-GFP  
                                         N-Term-GFP

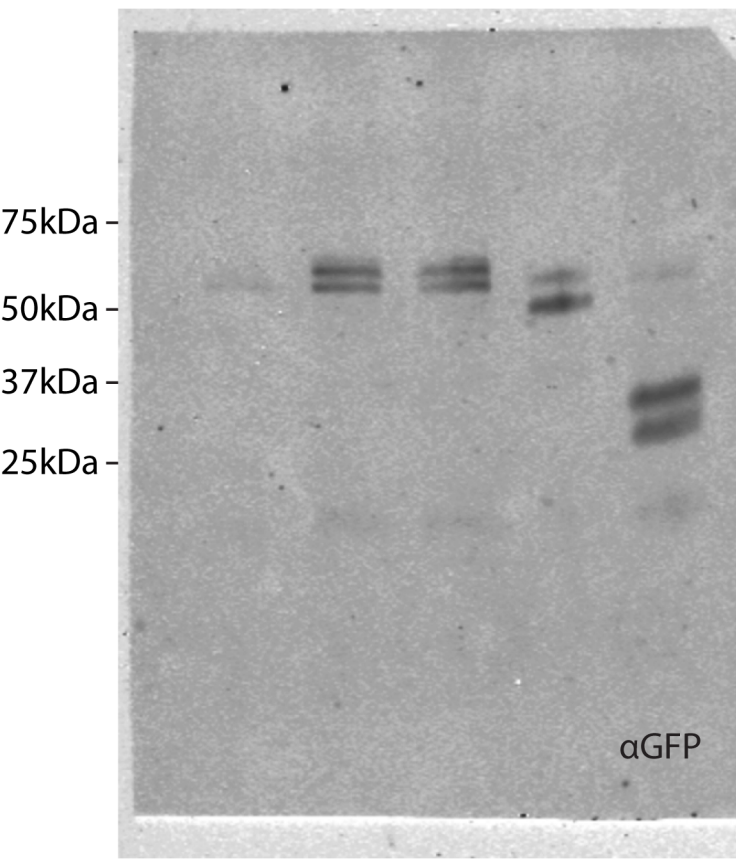

Unlabeled raw blot

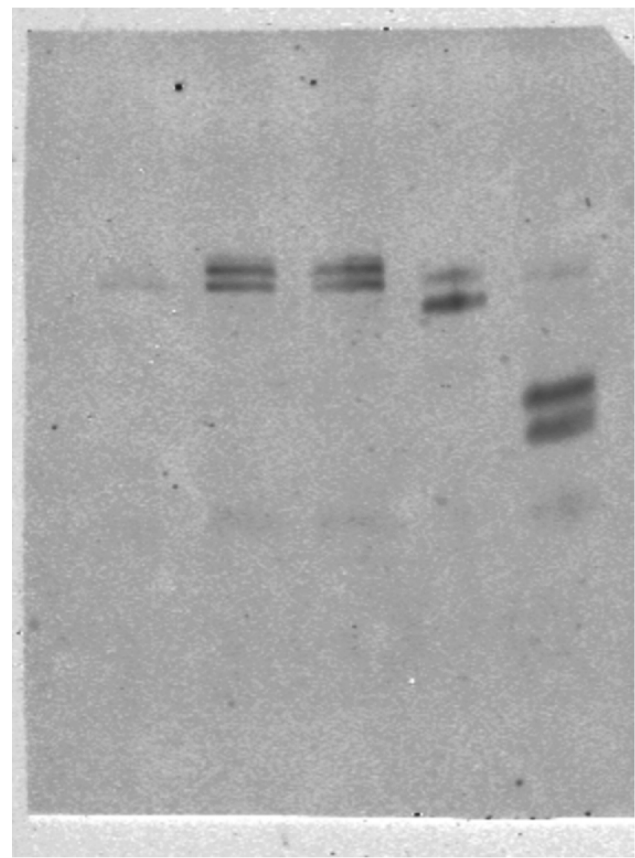

Supplement: Figure 4—figure supplement 3—source data 1. [file elife-100256-fig4-figsupp3-data1.zip › Figure 4 - source data 3 - episomal WB.pdf]

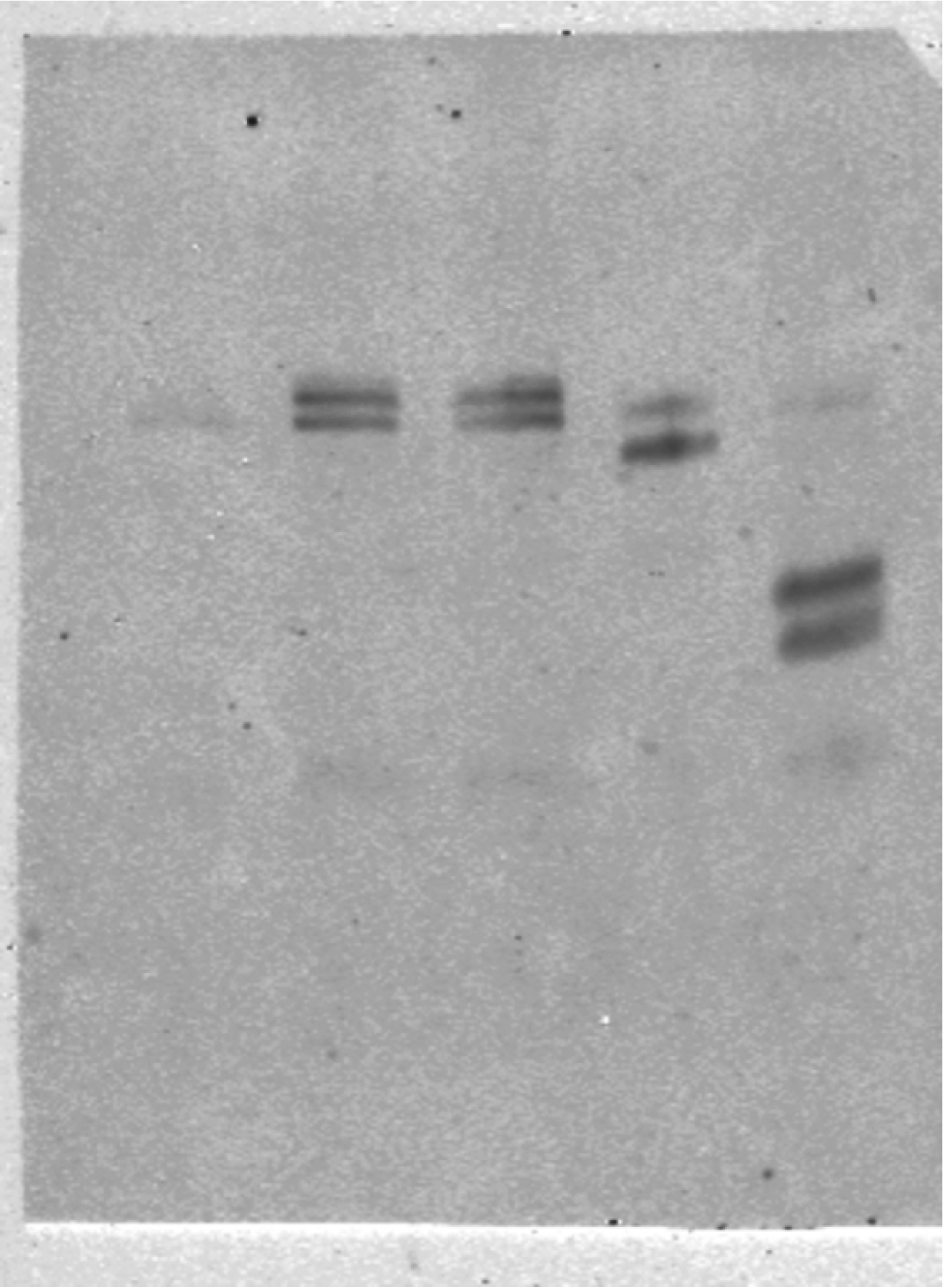

Supplement: Figure 4—figure supplement 3—source data 2. [file elife-100256-fig4-figsupp3-data2.zip › Figure 4 - figure supplement 3 - source data 1 - episomal WB unlabeled raw.tif]

Labeled gel

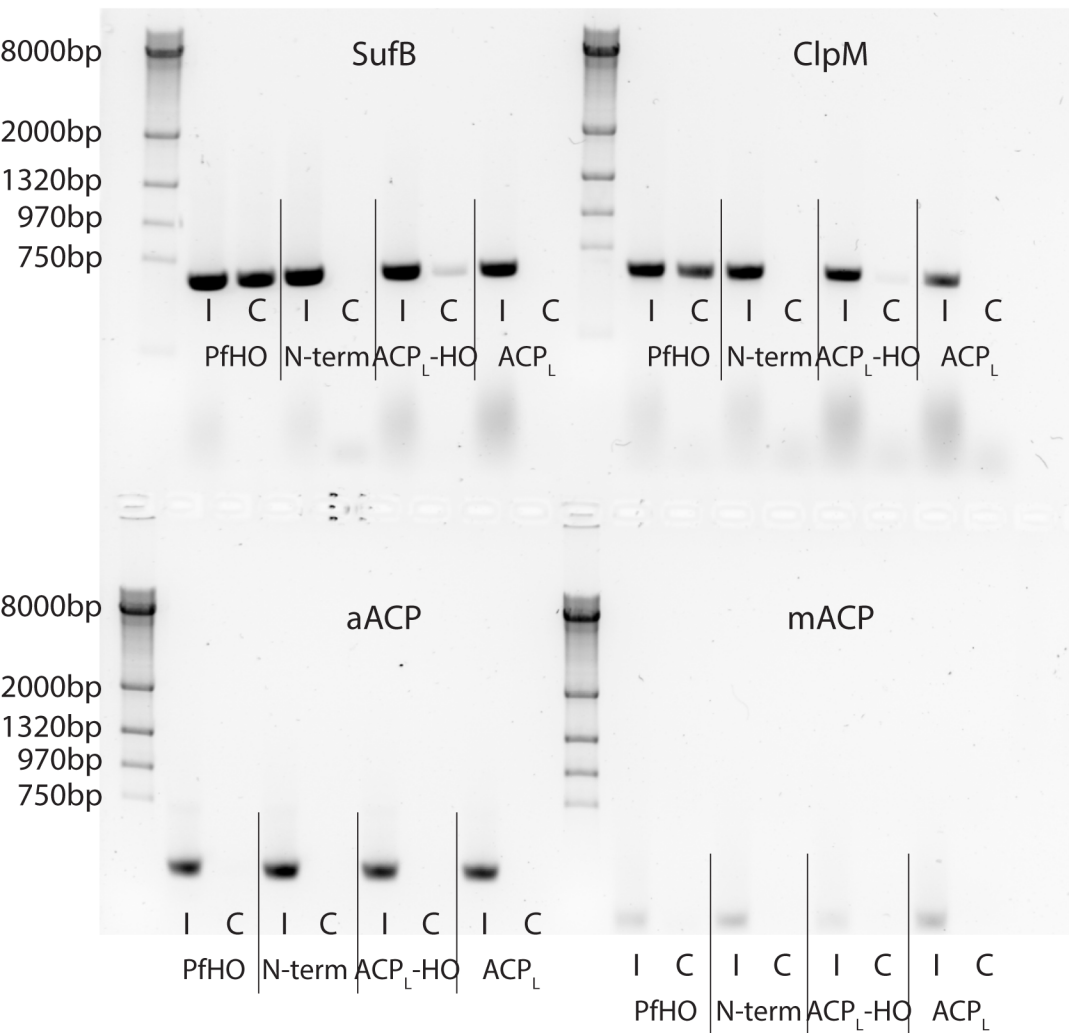

Unlabeled raw gel

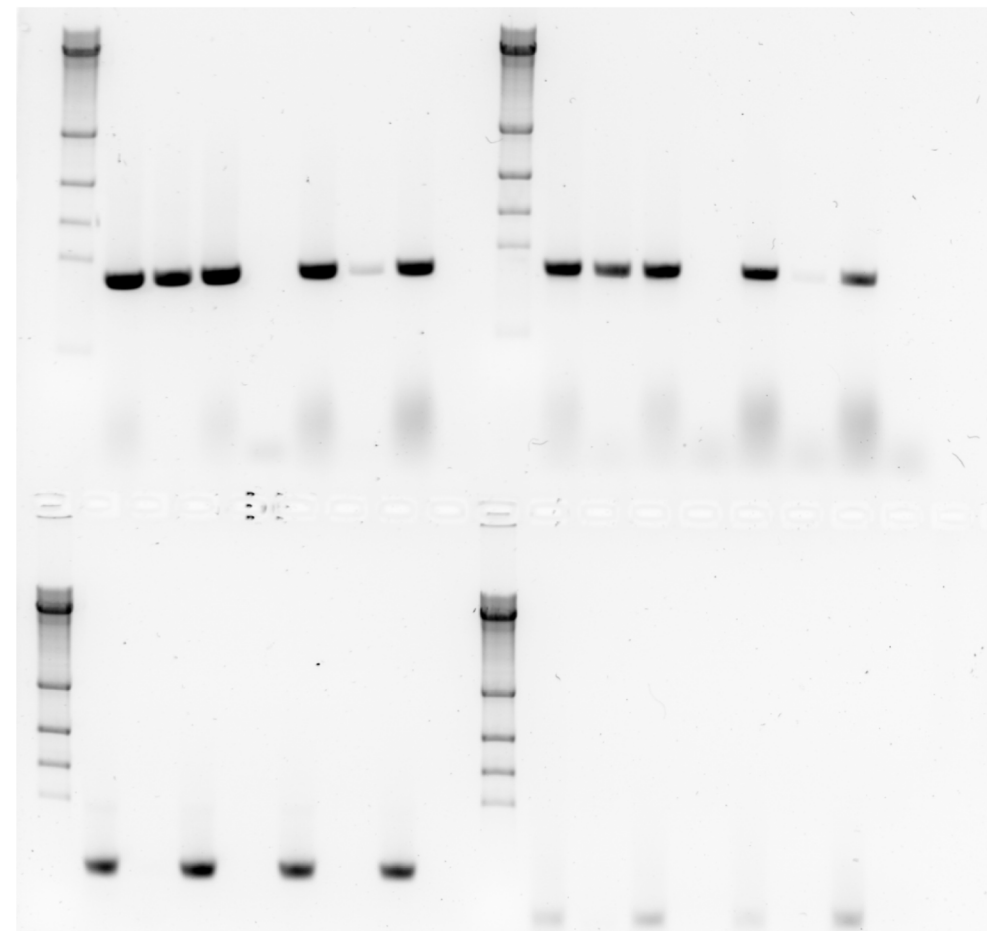

Supplement: Figure 5—source data 2. [file elife-100256-fig5-data2.zip › Figure 5 - source data 2 - Fig 5 and F5FS5.pdf]

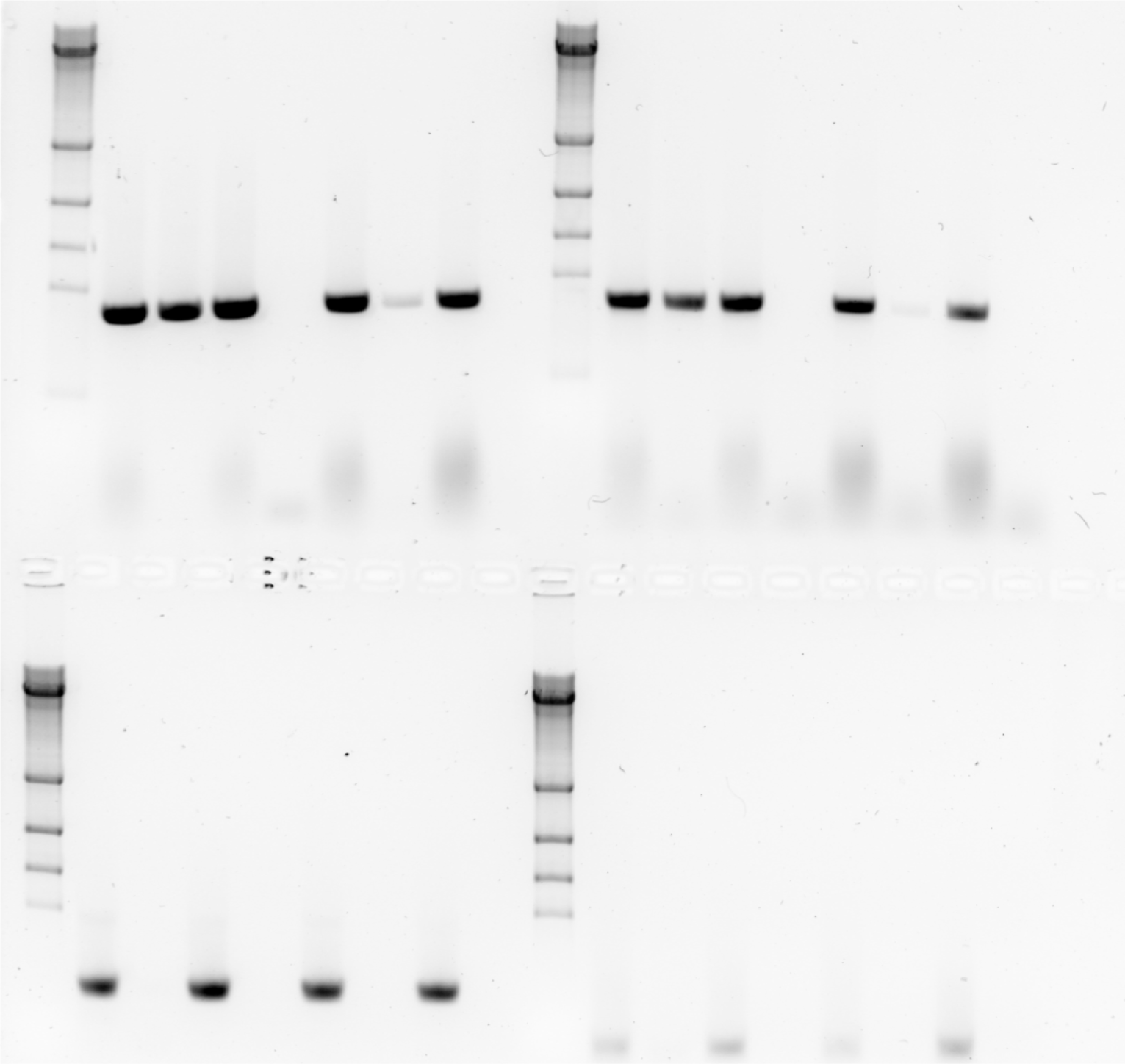

Supplement: Figure 5—source data 3. [file elife-100256-fig5-data3.zip › Figure 5 - source data 3 - F5 and F5FS5 unlabeled raw.tif]

Labeled gel

Unlabeled raw gel

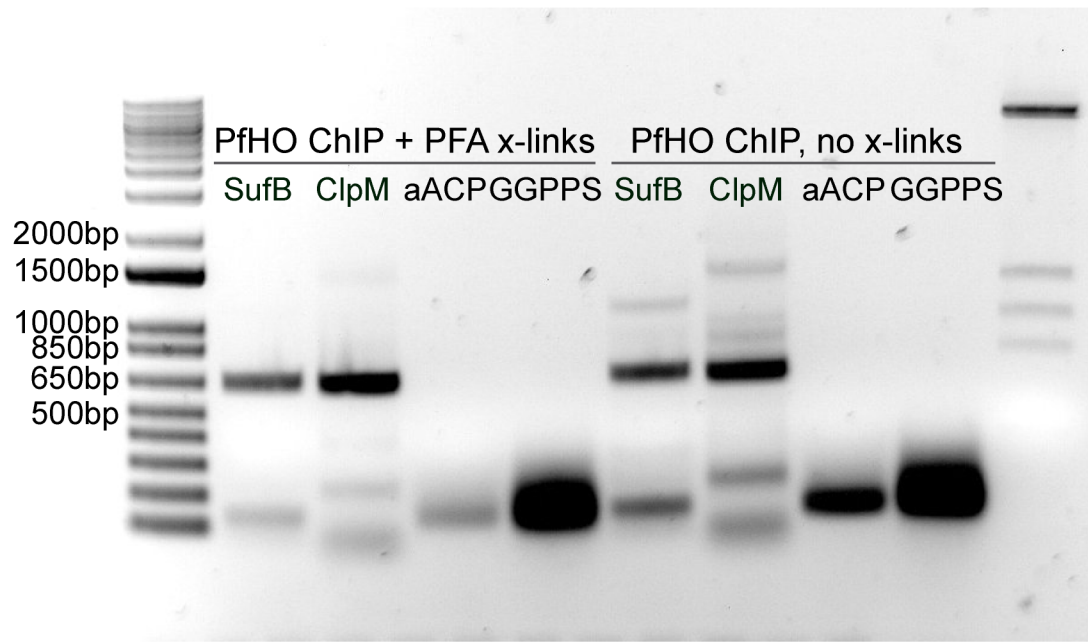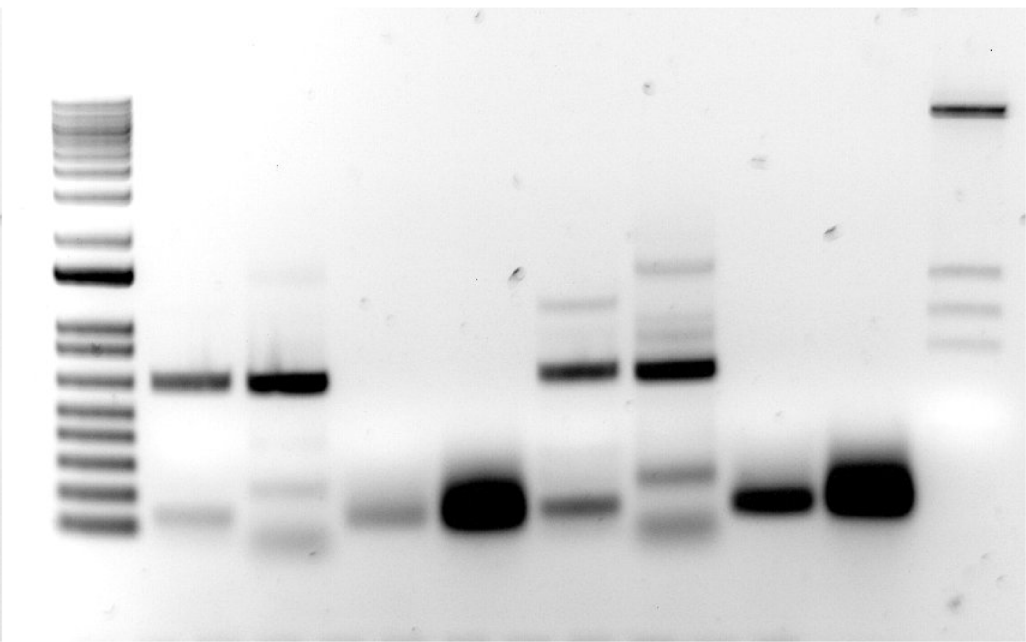

Supplement: Figure 5—figure supplement 6—source data 1. [file elife-100256-fig5-figsupp6-data1.zip › Figure 5 - source data 4 - ChIP xlinks gel.pdf]

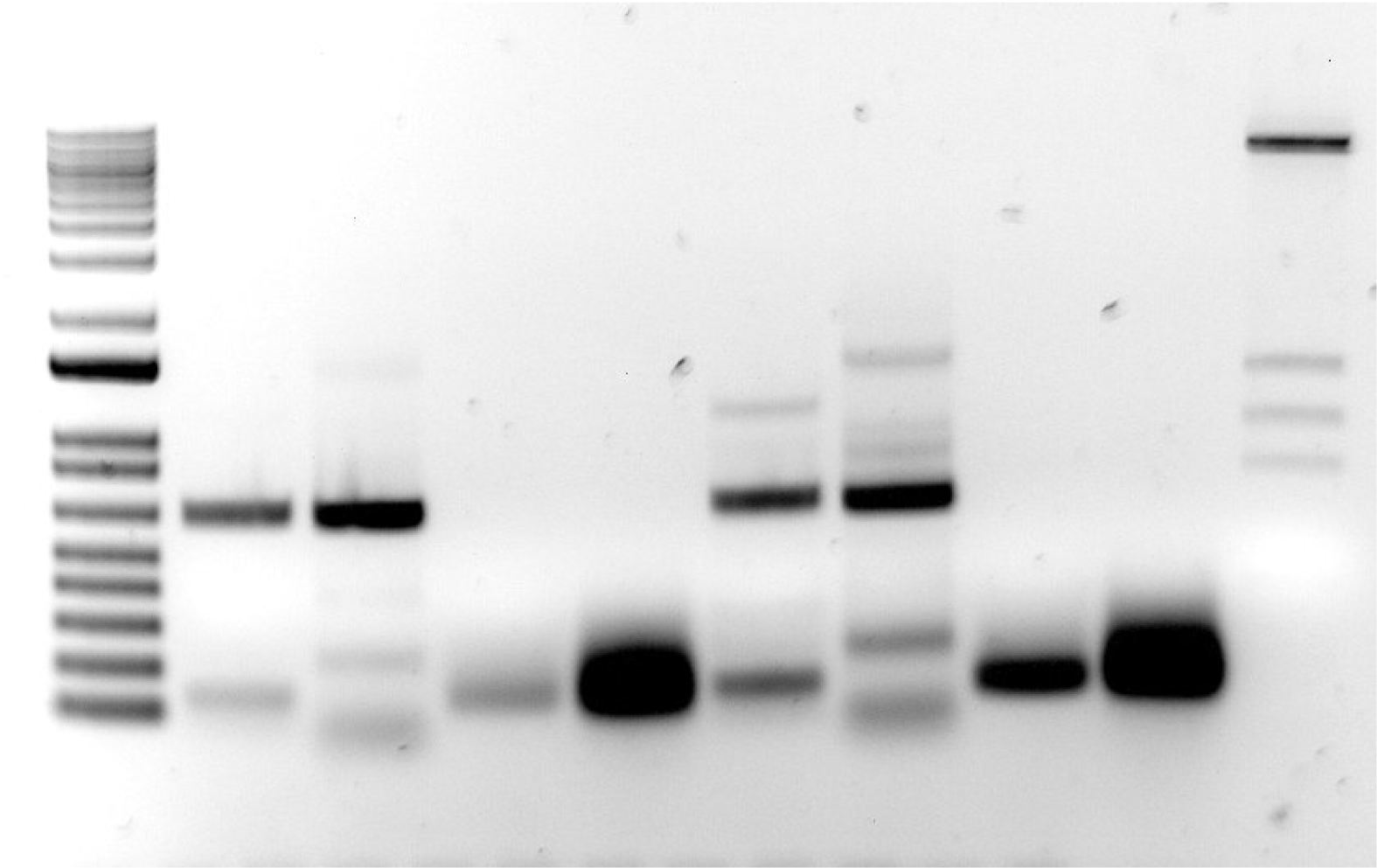

Supplement: Figure 5—figure supplement 6—source data 2. [file elife-100256-fig5-figsupp6-data2.zip › Figure 5 - source data 5 - ChIP xlinks gel unlabeled raw.tif]
