# Supplementary material for: Malaria parasites require a divergent heme oxygenase for apicoplast gene expression and biogenesis
Source: eLife. 2024 Dec 11;13:RP100256. doi: 10.7554/eLife.100256 (PMC11634067; doi:10.7554/eLife.100256)
Supplement: Figure 2—source data 3. [file elife-100256-fig2-data3.zip › figure 2 - source data 3 - NtermGFP WB.pdf]

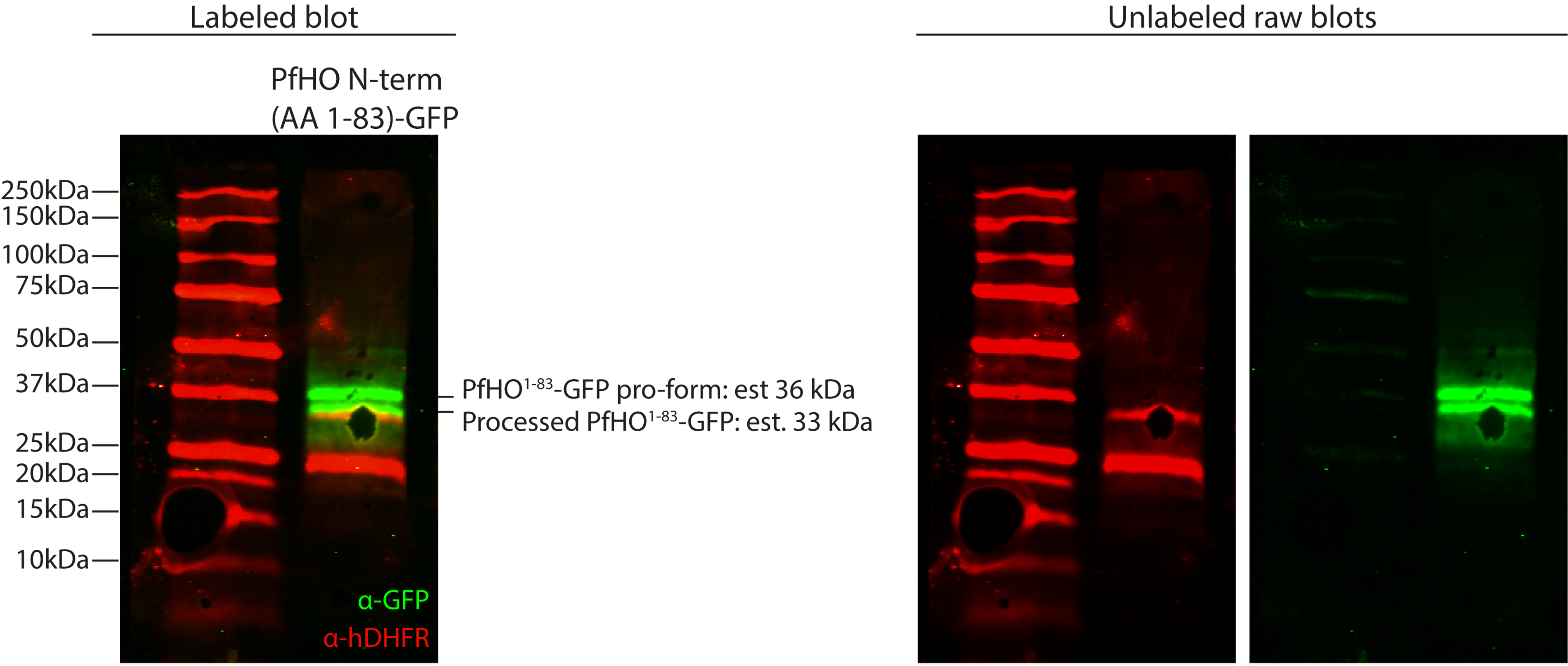
Labeled blot
Unlabeled raw blots
PfHO N-term
(AA 1-83)-GFP
250kDa
150kDa
100kDa
75kDa
50kDa
37kDa
25kDa
20kDa
15kDa
10kDa
PfHO1-83-GFP pro-form: est 36 kDa
Processed PfHO1-83-GFP: est. 33 kDa
α-GFP
α-hDHFR
