# Supplementary material for: Malaria parasites require a divergent heme oxygenase for apicoplast gene expression and biogenesis
Source: eLife. 2024 Dec 11;13:RP100256. doi: 10.7554/eLife.100256 (PMC11634067; doi:10.7554/eLife.100256)
Supplement: Figure 3—figure supplement 1—source data 3. [file elife-100256-fig3-figsupp1-data3.zip › figure 3 - source data 7 - PfHO-glmS PCR gel.pdf]

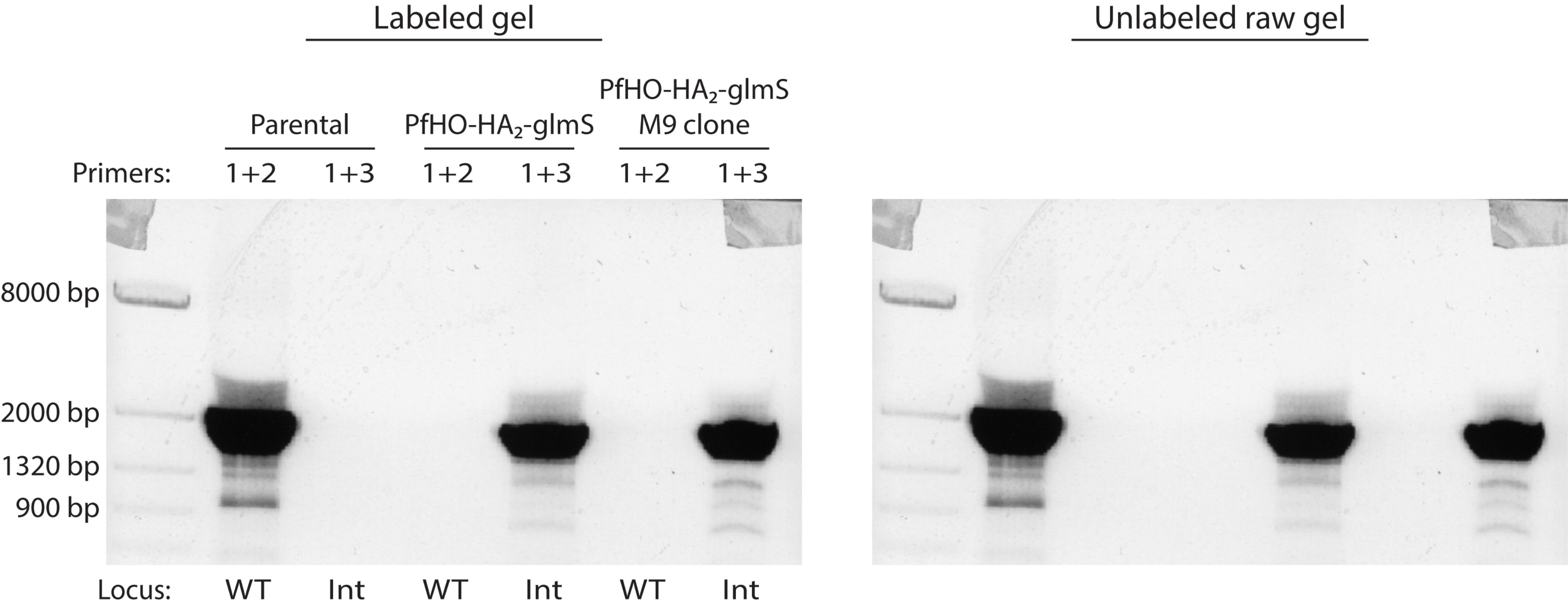

Labeled gel
Unlabeled raw gel
Parental
PfHO-HA₂-glmS
PfHO-HA₂-glmS M9 clone
Primers:
1+2
1+3
1+2
1+3
1+2
1+3
8000 bp
2000 bp
1320 bp
900 bp
Locus:
WT
Int
WT
Int
WT
Int
