# Supplementary material for: Malaria parasites require a divergent heme oxygenase for apicoplast gene expression and biogenesis
Source: eLife. 2024 Dec 11;13:RP100256. doi: 10.7554/eLife.100256 (PMC11634067; doi:10.7554/eLife.100256)
Supplement: Figure 3—figure supplement 5—source data 2. [file elife-100256-fig3-figsupp5-data2.zip › figure 3 - source data 15 - PfHO ab final bleed.pdf]

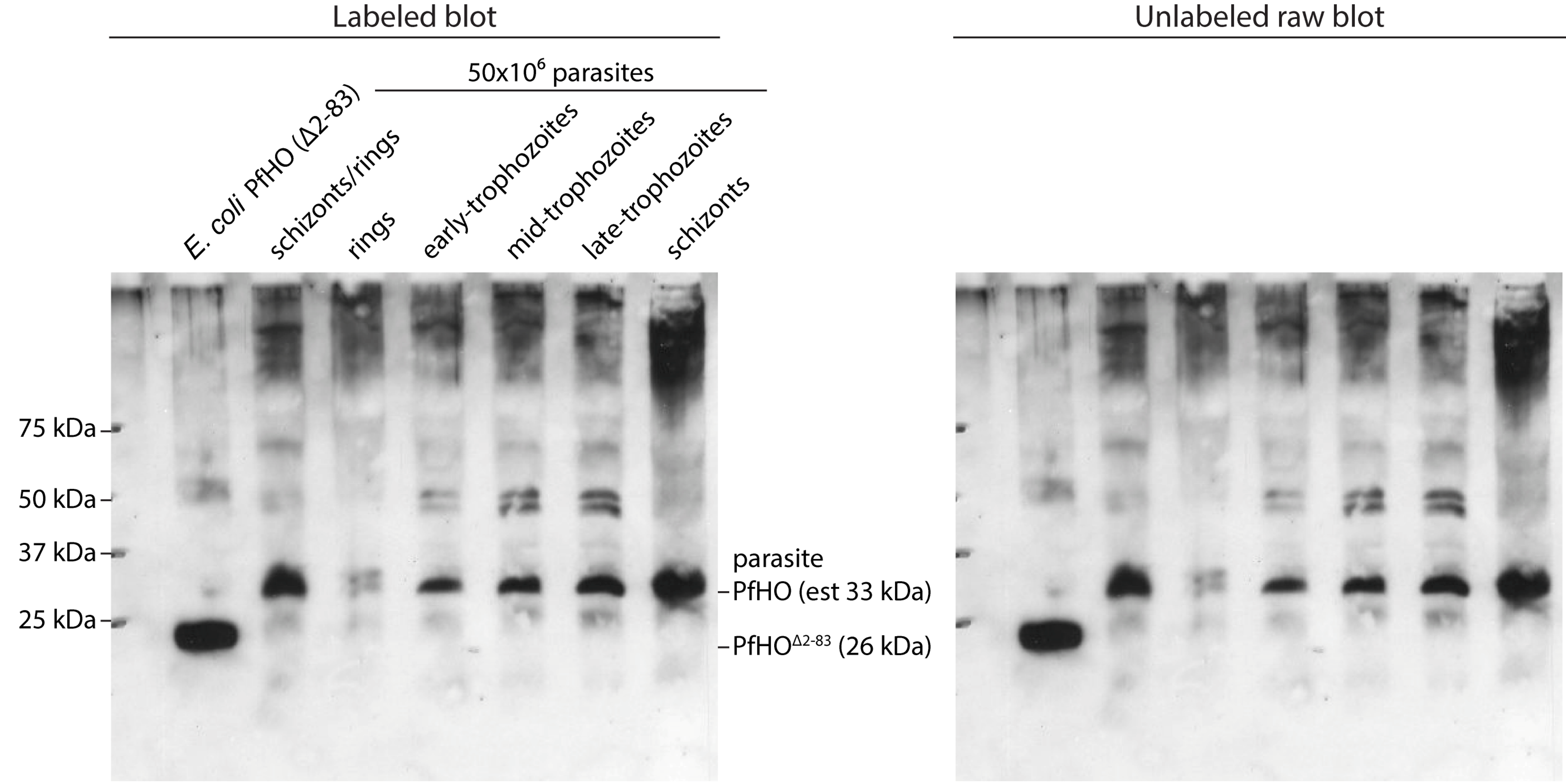

Labeled blot
Unlabeled raw blot
50x10^6 parasites
E. coli PfHO (Δ2-83)
schizonts/rings
rings
early-trophozoites
mid-trophozoites
late-trophozoites
schizonts
75 kDa
50 kDa
37 kDa
25 kDa
parasite
PfHO (est 33 kDa)
PfHO Δ2-83 (26 kDa)
