# Supplementary material for: Malaria parasites require a divergent heme oxygenase for apicoplast gene expression and biogenesis
Source: eLife. 2024 Dec 11;13:RP100256. doi: 10.7554/eLife.100256 (PMC11634067; doi:10.7554/eLife.100256)

Labeled blots

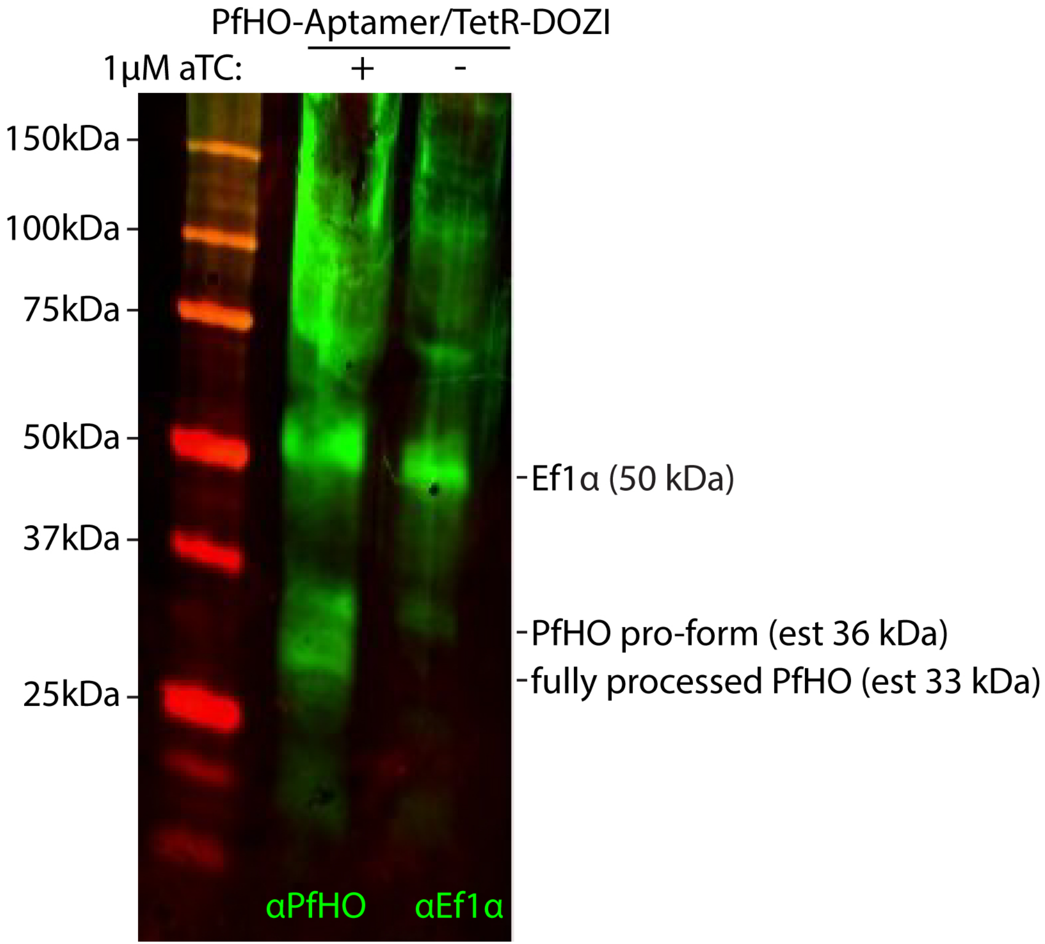

Unlabeled raw blots

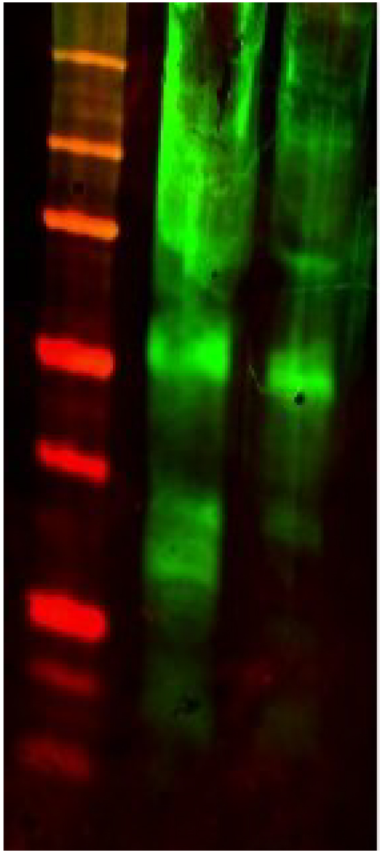

Supplement: Figure 3—figure supplement 6—source data 1. [file elife-100256-fig3-figsupp6-data1.zip › figure 3 - source data 17.pdf]
