# Supplementary material for: Malaria parasites require a divergent heme oxygenase for apicoplast gene expression and biogenesis
Source: eLife. 2024 Dec 11;13:RP100256. doi: 10.7554/eLife.100256 (PMC11634067; doi:10.7554/eLife.100256)
Supplement: Figure 4—source data 1. [file elife-100256-fig4-data1.zip › figure 4 - source data 1 - processing WB.pdf]

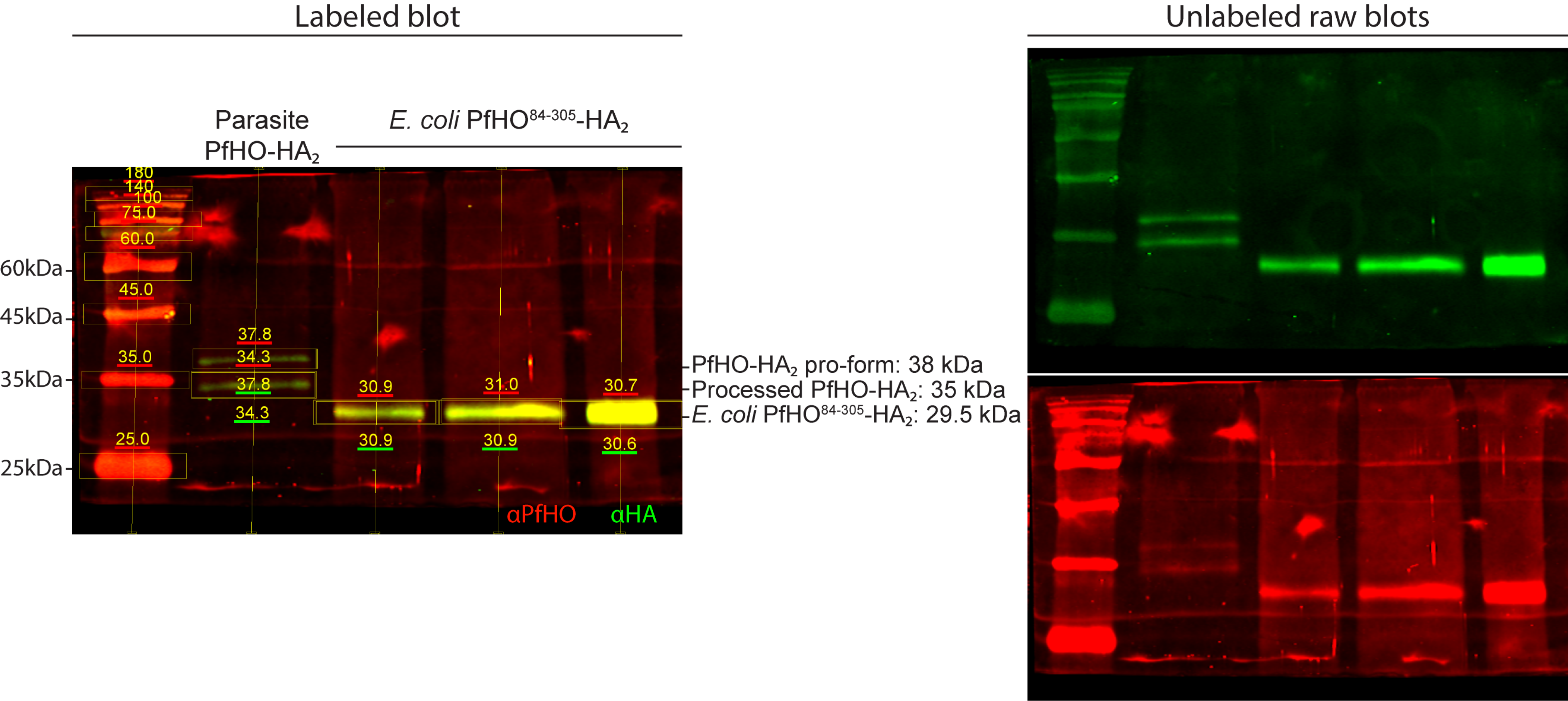
Labeled blot
Unlabeled raw blots
Parasite PfHO-HA2
E. coli PfHO84-305-HA2
180
140
100
75.0
60.0
45.0
35.0
25.0
60kDa
45kDa
35kDa
25kDa
37.8
34.3
37.8
34.3
30.9
31.0
30.7
30.9
30.9
30.6
PfHO-HA2 pro-form: 38 kDa
Processed PfHO-HA2: 35 kDa
E. coli PfHO84-305-HA2: 29.5 kDa
αPfHO
αHA
