# Supplementary material for: Malaria parasites require a divergent heme oxygenase for apicoplast gene expression and biogenesis
Source: eLife. 2024 Dec 11;13:RP100256. doi: 10.7554/eLife.100256 (PMC11634067; doi:10.7554/eLife.100256)
Supplement: Figure 4—figure supplement 1—source data 1. [file elife-100256-fig4-figsupp1-data1.docx]

**PfHO N-Term (1-83):**

**MIRKIIILMFTFFSNIHNEKIYHHKQRRKFLKGPLGYLNRNVIQKKHYNLYAKKFINYKEIQIQRINDYRKRSGVDKNNINYNLR**

**PfHO HO-domain (84-305):**

**DTYNYHETHLFVRNEVLPTLAKIENENLKEKEKNKEIFRNINDYNSNFTRQTFLQFLMDLYNIFLKIDDLFLENKTYFSILIYNGPMQLTNHLYDDIIYVSSVVENSDDVSPSEYCMEYISHLENLCEENKLSFLAHAYVFYKNFHLSKEHLLKSICKYLNIIKKLKSSTYVADVENFEFCLNKMSRKWSRWEKDNFLASLHNATNKMMILTKHFEKVKS**

**Peptides in PfHO N-Term (1-83)**

**K.GPLGYLNR**

**K.HYNLYAK**

**K.KFINYK**

**K.FINYK**

**F.INYKEIQIQRINDYR**

**R.INDYR**

**R.SGVDKNNINYNLR**

**K.NNINYNLR**

**Peptides in PfHO HO-domain (84-305)**

**R.DTYNYHETHLFVR**

**Y.NYHETHLFV**

**R.NEVLPTLAK**

**K.IENENLK**

**K.IENENLKEK**

**F.RNINDYNSNFT**

**R.NINDYNSNFTR**

**K.IDDLFLENK**

**F.LENKTYF**

**L.TNHLYDDIIYV**

**Y.VSSVVENSDDVSPSEYC**

**Y.VFYKNFH**

**L.KSICKYL**

**K.YLNIIK**

**K.SSTYVADVENFEFCLNK**

**Y.VADVENFEFC**

**R.WEKDNFLASLHNATNK**

**K.DNFLASLHNATNK**

**K.MMILTK**
